# rs822336 binding to C/EBPβ and NFIC modulates induction of PD-L1 expression and predicts anti-PD-1/PD-L1 therapy in advanced NSCLC

**DOI:** 10.1186/s12943-024-01976-2

**Published:** 2024-03-25

**Authors:** Giovanna Polcaro, Luigi Liguori, Valentina Manzo, Annalisa Chianese, Giuliana Donadio, Alessandro Caputo, Giosuè Scognamiglio, Federica Dell’Annunziata, Maddalena Langella, Graziamaria Corbi, Alessandro Ottaiano, Marco Cascella, Francesco Perri, Margot De Marco, Jessica Dal Col, Giovanni Nassa, Giorgio Giurato, Pio Zeppa, Amelia Filippelli, Gianluigi Franci, Fabrizio Dal Piaz, Valeria Conti, Stefano Pepe, Francesco Sabbatino

**Affiliations:** 1 Oncology Unit, Department of Medicine, Surgery and Dentistry, University of Salerno, Baronissi, 84081 Italy; 2 Oncology Unit, Department of Medicine, Surgery and Dentistry, University of Naples “Federico II”, Naples, 80131 Italy; 3 Clinical Pharmacology Unit, Department of Medicine, Surgery and Dentistry, University of Salerno, Baronissi, 84081 Italy; 4 University Hospital “San Giovanni di Dio e Ruggi d’Aragona”, Salerno, 84131 Italy; 5 Department of Experimental Medicine, University of Campania “Luigi Vanvitelli”, Naples, 80138 Italy; 6 Department of Medicine, Surgery and Dentistry, University of Salerno, Baronissi, 84081 Italy; 7 Pathology Unit, Department of Medicine, Surgery and Dentistry, University of Salerno, Baronissi, 84081 Italy; 8 Pathology Unit, Istituto Nazionale Tumori IRCCS Fondazione G. Pascale, Naples, 80131 Italy; 9 Hematology and Transplant Unit, University Hospital “San Giovanni di Dio e Ruggi d’Aragona”, Salerno, 84131 Italy; 10 Department of Translational Medical Sciences, University of Naples “Federico II”, Naples, 80131 Italy; 11 Division of Innovative Therapies for Abdominal Metastases, Istituto Nazionale Tumori IRCCS Fondazione G. Pascale, Naples, 80131 Italy; 12 Unit of Anesthesiology, Intensive Care Medicine, and Pain Medicine, Department of Medicine, Surgery and Dentistry, University of Salerno, Baronissi, 84081 Italy; 13 Medical and Experimental Head and Neck Oncology Unit, Istituto Nazionale Tumori IRCCS Fondazione G. Pascale, Naples, 80131 Italy; 14 Laboratory of Molecular Medicine and Genomics, Department of Medicine, Surgery and Dentistry, University of Salerno, Baronissi, 84081 Italy; 15 Clinical Microbiology Unit, Department of Medicine, Surgery and Dentistry, University of Salerno, Baronissi, 84081 Italy

**Keywords:** Immunotherapy, NSCLC, PD-1, PD-L1, Predictive biomarker, SNP, rs822336

## Abstract

Efficient predictive biomarkers are needed for immune checkpoint inhibitor (ICI)-based immunotherapy in non-small cell lung cancer (NSCLC). Testing the predictive value of single nucleotide polymorphisms (SNPs) in programmed cell death 1 (*PD-1*) or its ligand 1 (*PD-L1*) has shown contrasting results. Here, we aim to validate the predictive value of *PD-L1* SNPs in advanced NSCLC patients treated with ICIs as well as to define the molecular mechanisms underlying the role of the identified SNP candidate. rs822336 efficiently predicted response to anti-PD-1/PD-L1 immunotherapy in advanced non-oncogene addicted NSCLC patients as compared to rs2282055 and rs4143815. rs822336 mapped to the promoter/enhancer region of *PD-L1*, differentially affecting the induction of *PD-L1* expression in human NSCLC cell lines as well as their susceptibility to HLA class I antigen matched PBMCs incubated with anti-PD-1 monoclonal antibody nivolumab. The induction of PD-L1 expression by rs822336 was mediated by a competitive allele-specificity binding of two identified transcription factors: C/EBPβ and NFIC. As a result, silencing of C/EBPβ and NFIC differentially regulated the induction of PD-L1 expression in human NSCLC cell lines carrying different rs822336 genotypes. Analysis by binding microarray further validated the competitive allele-specificity binding of C/EBPβ and NFIC to *PD-L1* promoter/enhancer region based on rs822336 genotype in human NSCLC cell lines. These findings have high clinical relevance since identify rs822336 and induction of PD-L1 expression as novel biomarkers for predicting anti-PD-1/PD-L1-based immunotherapy in advanced NSCLC patients.

**Supplementary Information:**

The online version contains supplementary material available at 10.1186/s12943-024-01976-2.

## Background

Non-small cell lung cancer (NSCLC) is the leading cause of cancer-related death worldwide with an average 5-year survival rate of 18% [1]. Current treatment options for advanced NSCLC patients include chemotherapy, targeted therapy and immunotherapy. Platinum-based chemotherapy still plays an important role but, so far, it provides a modest clinical benefit [2]. Targeted therapy with tyrosine kinase inhibitors (TKIs) in addition to other types of small molecules, has led to a major advance in the management of NSCLC patients. Nevertheless, this type of therapy is only applicable to the small subset of patients who carry specific actionable oncogene alterations, defined as oncogene addicted [3–15]. Recently, immunotherapy with immune checkpoint inhibitors (ICIs) such as programmed cell death-1 (PD-1)- and programmed death-ligand 1 (PD-L1) monoclonal antibodies (mAbs) has revolutionized the treatment algorithm of several types of cancer including NSCLC [16–23]. In advanced non-oncogene addicted NSCLC, ICIs with or without chemotherapy have clearly shown to significantly improve progression-free survival (PFS) and overall survival (OS) of treated patients as compared to standard chemotherapy [18–20, 22, 24–27]. However, the efficacy of this novel immunotherapeutic strategy is limited to up to 40% of treated patients and there is still the need to identify predictive biomarkers of treatment response. Several biomarkers have been investigated including PD-L1 tumor expression, tumor mutational burden (TMB), human leukocyte antigen (HLA) class I and II expression, β2-microglobulin (β2m) mutations, tumor microenvironment (TME) composition, gene expression profiles (GEPs) and gut microbiome composition [28–31]. So far, PD-L1 expression by both cancer and intra-tumoral immune cells is the most widely explored. It is currently utilized to select patients who are more likely to benefit from anti-PD-1/PD-L1-based immunotherapy. Indeed an increased expression of PD-L1 by both cancer and intra-tumoral immune cells is associated with an increased likelihood of response to anti-PD-1/PD-L1-based immunotherapy [26, 27, 30, 32]. Nevertheless, not all high PD-L1 positive tumors respond to the treatment while some PD-L1 negative tumors benefit too [29, 30, 32, 33]. As a result, PD-L1 expression is currently not considered an efficient predictive biomarker [29, 30, 32, 33].

Genetic variants can influence multiple aspects of different types of disease including cancer. To date, over seventy thousand genetic variants (mainly, single nucleotide polymorphisms (SNPs)) have been associated with specific cancer features, survival outcomes, treatment response and drug related toxicity [34–36]. These genetic variants are mostly located in non-coding regions of DNA such as gene promoters or enhancers. The latter represent binding sites for transcription factors (TFBSs) that regulate gene expression [37–39]. As a result, SNPs localized in TFBSs can affect transcription factor (TF) binding affinity that in turn modifies target gene expression [37–39]. In the present study we investigated the predictive value of *PD-L1* SNPs in advanced NSCLC patients treated with ICIs, defining the molecular mechanisms of SNP mediated gene expression changes.

## Materials and methods

### Study population

Two cohorts of patients were included. In the first, Caucasian patients with confirmed advanced NSCLC were recruited from July 2017 to December 2021 at “San Giovanni di Dio e Ruggi D’Aragona” University Hospital. The study was performed without interfering with clinical practice. Selection of patients to be included in the study was performed based on: (i) signed informed consent for clinical-pathological data acquisition; (ii) age > 18 years; (iii) treatment with anti-PD-1/PD-L1 therapy as a second line following platinum-based chemotherapy failure; (iv) treatment with baseline prednisone equivalent dose ≤ 10 mg/day; (iv) absence of symptomatic brain metastases; (v) informed consent for blood sample collection and analysis. Patients with epidermal growth factor receptor (*EGFR*), anaplastic lymphoma kinase (*ALK*) or *ROS1* tumor alterations were excluded from the study. Evaluation of *EGFR*, *ALK* and *ROS1* alterations was performed on tumor samples (when available) or liquid biopsy according to national pathology guidelines. Clinical-pathological characteristics including age, sex, Eastern Cooperative Oncology Group (ECOG) performance status (PS), smoking status, comorbidities, tumor histology, presence/absence of asymptomatic brain metastases, were collected. After collecting individual data, response rate, median PFS, median OS and rate of immune-related adverse events (irAEs) were calculated. Patient privacy and personal data were preserved by assigning a progressive anonymous identification number. PD-L1 scoring was performed on tumor samples as clinically indicated when tumor tissue was available. PD-L1 was reported as tumor proportion score (TPS) [20] according to European Society for Medical Oncology (ESMO) guidelines. Patients received anti-PD-1 (nivolumab or pembrolizumab) or anti-PD-L1 (atezolizumab) mAbs according to ESMO guidelines. irAEs were reported according to Common Terminology Criteria for Adverse Events (CTCAE) v 4.0 [40]. Radiographic imaging was performed every 8 weeks. Response rate was determined according to Response Evaluation Criteria in Solid Tumours version 1.1 (RECIST v1.1) [41] and reported as complete response (CR), partial response (PR), stable disease (SD) and progressive disease (PD). Objective response rate (ORR) was defined as the proportion of patients with a CR or PR. PFS was defined as the time from the start of treatment to the first documented PD or death by any cause. OS was defined from the start of treatment to death by any cause or last follow-up date. Patients died from COVID-19 were excluded from the study. In the second cohort, Caucasian patients with confirmed advanced NSCLC, expressing PD-L1 < 50% (TPS), were recruited from April 2021 to September 2023 at same Institution. All patients were treated as first line with anti-PD-1 pembrolizumab and platinum-based chemotherapy, according to clinical practice. Patients with *EGFR*, *ALK, ROS1, BRAF V600*, neurotrophic tyrosine receptor kinase 1 and 2 (*NTRK1* and *2*), rearranged during transfection (*RET)*, mesenchymal epithelial transition factor (*MET*) and human epidermal growth factor receptor 2 (*HER2*) tumor alterations were excluded from the study. The study was approved by the local ethics committee (prot./SCCE n.85,275), in accordance with the Declaration of Helsinki and its amendments.

### Chemical reagents and antibodies

Interferon gamma (IFN-γ) was purchased from PeproTech EC (London, UK). Nivolumab was purchased from Thermo Fisher (Waltham, MA, USA). mAb LGIII-147.4.1 which recognizes β2m-associated HLA-A heavy chain, excluding -A23, -A24, -A25, -A32 [42]; mAb B1.23.2 which recognizes β2m-associated HLA-B and -C heavy chains [43]; mAb TP25.99.8.4 which recognizes β2m-associated HLA-A, -B and -C heavy chains [44]; mAb NAMB-1 which recognizes β2-microglobulin [45]; mAb LGII-612.14 which recognizes HLA-DR, DQ, DP [46] and anti-idiotypic (anti-id) mAb MK2-23 [47] were all kindly provided from Prof. Soldano Ferrone (Massachusetts General Hospital, Harvard Medical School, Boston, MA, USA). Allophycocyanin (APC) anti-PD-L1 IgG2b Ab (catalog on. 329,708), APC anti-mouse IgG2b Ab (catalog on. 406,712) and purified human IgG4 isotype control (catalog on. 403,402) were purchased from BioLegend (San Diego, CA, USA). Fluorescein isothiocyanate (FITC) anti-mouse IgG Ab (catalog on. 115-095-146) was purchased from Jackson Immuno Research (Philadelphia, PA, USA). PD-L1- (catalog on. 13,684), STAT1- (catalog on. 14,994), p-STAT1- (catalog on. 9167), GAPDH- (catalog on. 5174), NFIC- (catalog on. 11,911) specific Abs, horseradish peroxidase (HRP) anti-rabbit Ab (catalog on. 7074), HRP anti-mouse Ab (catalog on. 7076) were purchased from Cell Signaling (Danvers, MA, USA). C/EBPβ specific Ab (catalog on. GTX100675) was purchased from Genetex (Irvine, CA, USA). FITC anti-CD107a (LAMP-1) IgG1 Ab (catolog on. BD555800) was purchased from BD Pharmingen (San Jose, CA, USA). Polymeric nanomatrix conjugated to humanized CD3 and CD28 agonist (T Cell TransAct human; catalog on. 130-111-160) was purchased from Miltenyi Biotec (Bergisch Gladbach, Germany).

### *PD-L1* SNP genotyping

Three previously reported *PD-L1* SNPs related to autoimmunity, cancer predisposition and cancer prognosis were selected to be genotyped [48–50]. PBMCs were obtained from NSCLC patients before starting treatment with anti-PD-1/PD-L1 therapy and isolated as described [51]. Isolated PBMCs were stored at -80 °C. *PD-L1* SNPs were genotyped from DNA extracted from both isolated PBMCs and NSCLC cell lines (HCC827, H1975, PC-9, H460, A549, H1299, H1703, and H1437) utilizing the Maxwell® 16 blood DNA purification kit (Promega, Madison, WI, USA) according to the manufacturer’s protocol. Quantity and quality of purified DNA were assessed in every sample using a NanoDrop spectrophotometer (Thermo Scientific, Wilmington, DE, USA). Genotyping of *PD-L1* SNPs was performed using TaqMan genotyping assay (*PD-L1* gene: C_1348559_10 for rs822336, C_1409286_1 for rs2282055 and C_31941235_10 for rs4143815) as described [52].

### HLA class I typing

HLA class I genotyping on NSCLC cell lines and PBMCs isolated from healthy donors was performed at the Transplant Hematological Unit of “San Giovanni di Dio e Ruggi D’Aragona” University Hospital. Specifically, PBMCs were obtained from healthy donors following informed consent acquisition for blood sample collection and isolated as described [51]. Genomic DNA was obtained from all nucleated cell using QIAamp DNA Mini Kit (Qiagen). After DNA quantification using a BioSpectrometer (Eppendorf, Hamburg, Germany), the concentration of DNA was adjusted to 50–150 ng/µl. HLA typing was carried out in accordance with the European Federation of Immunogenetics (EFI) international standards using molecular genetic typing methods. HLA typing was performed at the basic resolution level. Allele groups of HLA genes (loci A, B, C) were determined using polymerase chain reaction (PCR) with sequence-specific primers (SSP) approved for clinical use produced by Protrans (Hockenheim, Germany) according to manufacturers’ protocol. The primer sets amplified the alleles described by the WHO international nomenclature committee (http://www.anthonynolan.org.uk/HIG/index.html). The primer mixes also contain a non-allelic amplification control primer pair which amplifies either a part (89 bp) of the beta-globin gene (HLA class I) or a part (440 bp) of the C-reactive-protein gene (HLA class II). The amplified DNA was visualized by agarose gel (2%) electrophoresis. The PCR amplification results in the gel were analyzed by the Software Helmberg SCORE. HLA class I genotyping of NSCLC cell lines was validated using TRON Cell Line Portal (http://celllines.tron-mainz.de/). Isolated PBMCs were cultured in RPMI 1640 medium (Euroclone, Milan, Italy) supplemented with 10% fetal bovin serum (FBS; Euroclone) and 1% penicillin-streptomycin (Euroclone), at 37 °C in a humidified incubator containing 5% CO_2_.

### Cell cultures

Human NSCLC cell lines carrying EGFR mutations (EGFR^mut^) (HCC827, H1975, PC-9) and EGFR wild type (EGFR^wt^) (H1299, H1703, H1437, H460 and A549) were obtained from American Type Culture Collection (ATCC; Manassas, VA, USA). Cancer cells were cultured in RPMI 1640 or DMEM medium (Euroclone, Milan, Italy) supplemented with 10% fetal bovin serum (FBS; Euroclone) and 1% penicillin-streptomycin (Euroclone). Cells were cultured at 37 °C in a humidified incubator containing 5% CO_2_. NSCLC cell lines were regularly tested for mycoplasma contamination with the use of a MycoAlert Mycoplasma Detection Kit (Lonza, Basel, Switzerland) following the manufacturer’s instructions.

### Flow cytometry analysis

NSCLC cell lines were seeded into 6-well plates at a density of 2 × 10^6^ cells per well and incubated with IFN-ɣ (100ng/ml). Untreated cells were used as a control. Following a 48 h incubation at 37 °C in a 5% CO_2_ atmosphere, cells were harvested and cell surface stained with either APC anti-PD-L1 IgG2b Ab or indicated HLA class I/II antigen- and β2m-specific mAbs. APC anti-mouse IgG2b and mAb MK2-23 were used as a specificity control for PD-L1 and HLA class I/II/β2m staining, respectively. HLA class I/II/β2m staining was detected by FITC anti-mouse IgG Ab. Staining was performed as described [51]. Stained cells were analyzed using a FACSVerse flow cytometer (BD Biosciences, Swindon, UK). Data, expressed as MFI, are representative of the results obtained in three independent experiments.

### cDNA synthesis and quantitative real-time (RT)-PCR

NSCLC cell lines were seeded into 24-well plates at a density of 2 × 10^5^ cells per well and incubated with IFN-ɣ (100ng/ml). Untreated cells were used as a control. Following a 24 h incubation at 37 °C in a 5% CO_2_ atmosphere, total RNA was extracted using TRIzol (Thermo Fisher) according to the manufacturer’s protocol. The quantity and quality of RNA samples were assessed using NanoDrop spectrophotometer (Thermo Scientific). cDNA was transcribed using the SensiFAST cDNA Synthesis Kit (Bioline, Memphis, TN, USA) according to the manufacturer’s protocol. Reverse transcription reactions were performed in a T100 Thermal Cycler (Bio-Rad Laboratories Inc., Hercules, CA, USA) according to the manufacturer’s protocol. mRNA expression levels of PD-L1, C/EBPβ and NFIC mRNA were evaluated by RT-PCR. GAPDH was used as internal reference gene. RT-PCR was performed in duplicate using SensiFAST SYBR No-ROX Kit (Bioline) according to the manufacturer’s protocol. PCR reactions were carried out in LightCycler 480 II (Roche, Basel, Switzerland). Primer sets were PD-L1fw: AAATGGAACCTGGCGAAAG, PD-L1rv: GCTCCCTGTTTGACTCCATC; C/EBPβ fw: GACAAGCACAGCGACGAGTA, C/EBPβ rv: AGCTGCTCCACCTTCTTCTG; NFICfw: GGACAGGGATGGGCTCTG, NFICrv: CGTTCTTCTGAGGCCAGTGC; GAPDHfw: CTGACTTCAACAGCGACACC, GAPDHrv: TAGCCAAATTCGTTGTCATACC. Gene expression profile analysis was performed using the 2-ΔΔCq method [53]. Data are representative of the results obtained in three independent experiments.

### Western blot analysis

NSCLC cell lines were seeded into 6-well plates at a density of 2 × 10^6^ cells per well and incubated with IFN-ɣ (100ng/ml). Untreated cells were used as a control. Following a 48 h incubation at 37 °C in a 5% CO_2_ atmosphere, cells were harvested and lysed as described [54]. Cell lysates were analyzed by western blot with PD-L1-, C/EBPβ - and NFIC-specific Abs. GAPDH was used as a loading control. Data are representative of the results obtained in three independent experiments.

### Cell morphology and trypan blue exclusion test

NSCLC cell lines were seeded into 6-well plates at a density of 2 × 10^6^ cells per well and incubated with IFN-ɣ (100ng/ml). Untreated cells were used as a control. Following a 48 h incubation at 37 °C in a 5% CO_2_ atmosphere, three random regions from both untreated and treated cells were captured (bars: 50 mm) with an inverted microscope Leica DM IL LED (Leica, Wetzlar, Germany). Cell viability was evaluated by 0.2% trypan blue exclusion test. Live cells were counted in duplicate with Automated Cell Counter (Thermo Fisher). Data are representative of the results obtained in three independent experiments.

### Co-culture of NSCLC cell lines with HLA-matched PBMCs

NSCLC cell lines were seeded into 24-well plates at a density of 2 × 10^5^ cells per well. HLA-matched PBMCs were activated for 1 h before co-culturing with an anti-CD3 (1 µg/mL) and anti-CD28 (1 µg/mL) T Cell Trans Act (T-Act). Following a 24 h incubation at 37 °C in a 5% CO_2_ atmosphere, PBMCs were added to cancer cells (10:1 ratio) and incubated with 10 µg/ml of nivolumab. The dose of nivolumab was selected based on anti-tumor activity tested in vitro on NSCLC cell lines [57]. Non-activated HLA-matched PBMCs were used as a control. Purified human IgG4 was used as a control for nivolumab.

### Cytotoxicity assays

Following a 48 h co-culture of cancer cells with HLA-matched PBMCs, cancer cells were isolated with phosphate-buffered saline (PBS) washing. Cancer cell viability was determined by cell counting kit-8 (CCK-8) assay (Dojindo Laboratories, Rockville, MD, USA) according to manufacturers’ protocol. In addition, following a 48 h co-culture of cancer cells with HLA-matched PBMCs, cancer cytotoxicity was determined from the harvested medium utilizing the Lactate Dehydrogenase (LDH) activity assay kit (Elabscience, Wuhan, Hubei, China) according to manufacturers’ protocol. The absorbances at 450 nm were determined by the Sunrise microplate reader (TECAN, Männedorf, Switzerland). Apoptosis induction was detected by annexin V and propidium iodide (PI; BD Bioscience) cytometric staining as described [55]. All experiments were performed in triplicate and repeated three times.

### PBMC cytotoxicity assays

Following a 48 h co-culture of cancer cells with HLA-matched PBMCs, the medium and PBMCs were harvested. IFN-γ and TNFα levels on harvested medium were analysed using ELISA Max Deluxe Set Human IFN-γ (BioLegend) and Human TNFα ELISA (Diaclone, Besancon Cedex, France) assays according to the manufacturers’ protocols. The absorbance at 450 nm was determined by the Sunrise microplate reader (TECAN, Männedorf, Switzerland). Degranulation assay on harvested PBMCs was performed utilizing CD107a staining by flow cytometry by analysing the percentages of stained cells. All experiments were performed in triplicate and repeated three times.

### In silico analysis

*PD-L1* SNPs were mapped using NCBI dbSNP (http://www.ncbi.nlm.nih.gov/SNP) and ENSEMBL v58 (http://www.ensembl.org/) databases. Analysis of putative TFs that bound to rs822336 was performed using TRANSFAC v6.4 bioinformatic tool by PROMO v3.0.2 software (https://alggen.lsi.upc.es). The sequences carrying each allele were loaded as the query sequence to search for potential TFs. The prediction included only human binding sites and TFs. The GO-based classification of the proteins identified in the DNA pull-down assay was performed using the PANTHER classification system (http://www.pantherdb.org/). The Cancer Genome Atlas (TCGA) data analysis was performed considering the dataset Lung adenocarcinoma (TCGA, Firehose Legacy) available in cBioPortal website (https://www.cbioportal.org/), that was used to obtain the list of samples with and without EGFR mutation status and to download the CD274 expression values associated to these samples. For selected samples, and where available, WGS data were downloaded from GDC data portal (https://portal.gdc.cancer.gov/) in bam format and converted in fastq format using samtools [56]. Raw sequences were aligned against human genome (assembly hg38) using BWA [57] with default parameters while the presence of the SNP rs822336 was assessed using VarScan2, setting the default parameters [58]. ENCODE Transcription Factor Targets was used to analyze target genes of TFs on DNA-binding by ChIP-seq (https://maayanlab.cloud/Harmonizome/dataset/ENCODE+Transcription+Factor+Targets).

### DNA pull-down assay and proteomic analyses by LC-MS/MS

NSCLC cell lines were seeded into T75 flasks at a density of 5 × 10^6^ cells. Nuclear extraction was performed as described [59]. Three different double-stranded oligos of 51 bp with 5’ biotinylated were designed by Primer3 v4.1.0 software (https://primer3.ut.ee/) and synthesized by Thermo Fisher (Supplementary file 1). The first set of primers consisted of the region containing rs822336 G +/- 25 bp (X-Y) in the forward oligo (wt). The second set of primers consisted of X-Y region containing a G→C mutation in the forward oligo (mut). The last one was a scrambled set of primers utilized as a control to exclude non-specific binding proteins. DNA pull-down procedure was performed for each biotinylated oligo with nuclear extract obtained from each cell line as described [59] utilizing (i) a DNA binding buffer containing HEPES pH 8.0 (10mM), NaCl (10mM), EDTA (10mM) and NP40 (0.05%); (ii) 500 µg of nuclear extract incubated with Streptavidin-coated beads (Dynabeads™ M-280, Thermo Fisher) according to the manufactures’ protocol; and (iii) a protein binding buffer containing HEPES pH 8.0 (50mM), NaCl (150mM), NP40 (0.1%), DTT (1mM) and complete protease inhibitors EDTA-free (Thermo Fisher). Obtained streptavidin-coated beads, binding the interactors of each oligo, were suspended in 10 mM ammonium bicarbonate containing 1.5 ng/µl of trypsin (Promega). The mixture was incubated at 37 °C overnight and the obtained peptides were subjected to NanoUPLC-hrMS/MS analyses as described [60].

### RNA interference transfection

A gene-specific package containing a siRNA-control and three preselected C/EBPβ (SR300760A, SR300760B, SR300760C) and NFIC (SR303158A, SR303158B, SR303158C) siRNAs were purchased from OriGene (Rockville, MD, USA; catalog on. SR300760 and SR303158, respectively). NSCLC cell lines were seeded into 6-well plates at a density of 2 × 10^6^ cells per well and transiently transfected with siRNAs using Lipofectamine RNAiMAX Transfection Reagent (Thermo Fisher) according to the manufacturer’s protocol. Following a 48 h transfection, cells were incubated with IFN-γ (100 ng/ml). Functional assays, RNA and protein analyses were performed within 24/48 h from transfection, respectively.

### Statistical analysis

All data were collected using Microsoft Excel. Statistical analyses were performed using STATA v13 software released by StataCorp LP (College Station, TX, USA) or GraphPad Prism v6.0 released by GraphPad Software (La Jolla, CA, USA). Continuous data were expressed as medians and ranges, whereas categorical data were expressed as frequencies and percentages. PFS and OS were calculated using the Kaplan-Meier method. Correlation between clinical-pathological characteristics and ORR, *PD-L1* SNPs or EGFR status was performed using the Fisher’s exact test, Mann–Whitney U test and the Kruskal-Wallis method, as appropriate. Correlation between clinical-pathological characteristics or *PD-L1* SNPs and survival outcomes (PFS and OS) was performed using log-rank test. Multivariate survival analyses were performed using the Cox proportional hazards model. The difference between groups was calculated using the two-sided, unpaired t test or one-way ANOVA. The difference between groups was considered significant when the *P* value was < 0.05.

## Results

### Clinical-pathological characteristics of NSCLC patients treated with ICIs

A total of 44 Caucasian patients with a confirmed diagnosis of advanced non-oncogene addicted NSCLC from the “San Giovanni di Dio e Ruggi D’Aragona” University Hospital were enrolled from July 2017 to December 2021. All patients were treated with second-line therapy with anti-PD-1/PD-L1 mAbs following platinum-based chemotherapy. Baseline medical record information including clinical-pathological characteristics of the patients are summarized in Table 1. Median age was 69 years (range, 48–83 years). Thirty-seven patients (84.00%) were male. Thirty-one (70.45%) and 13 (29.55%) had ECOG PS of 0–1 and 2, respectively. Two patients (4.55%) were never smokers, while 31 (70.45%) and 11 (25.00%) were previous and current smokers, respectively. Relevant comorbidities comprised hypertension (70.45%), dyslipidemia (38.64%), diabetes (25.00%), chronic obstructive pulmonary disease (COPD) (18.18%) and heart failure (13.64%). Twenty-five tumors (58.14%) were classified as adenocarcinomas while 18 (41.86%) were squamous NSCLC. Asymptomatic brain metastases were present in 8 (18.60%) patients. PD-L1 TPS was available for 23 patients (52.27%). A PD-L1 TPS ≥ 1% was reported in 82.61% of the analyzed tumors. Most patients (84.09%) were treated with anti-PD-1 mAbs. Specifically, twenty-eight (63.64%) and 9 (20.45%) were treated with anti-PD-1 nivolumab and pembrolizumab, respectively; seven (15.91%) patients were treated with anti-PD-L1 atezolizumab. The ORR was 22.73%. CRs, PRs, SDs and PDs were reported in 2 (4.55%), 8 (18.18%), 9 (20.45%) and 25 (56.82%) of treated patients, respectively. At a median follow-up of 41.46 months (range, 12.56–53.20 months) 8 out of 44 patients (18.18%) were still alive. Median PFS and OS were 3.52 months and 8.53 months, respectively (Fig. S1). Grade 1–2 and grade 3–4 immune-related adverse events (irAEs) were reported in 30 (69.77%) and 4 (9.3%) treated patients, respectively. No treatment-related deaths were reported.

**Table 1:** Baseline clinical-pathological characteristics of patients included in the study

Median age	69 years (48–83 years)
**Sex:**	
Male	37 (84.00%)
Female	7 (16.00%)
**ECOG PS**:	
0	12 (27.30%)
1	19 (43.20%)
2	13 (29.50%)
**Smoking status**:	
Never smoker	2 (4.55%)
Previous smoker	31 (70.45%)
Current smoker	11 (25.00%)
**Comorbidities**:	
Hypertension	31 (70.45%)
Dyslipidemia	17 (38.64%)
Diabetes	11 (25.00%)
COPD	8 (18.18%)
HF	6 (13.64%)
CRF	0 (0.00%)
Immune disorder	0 (0.00%)
**Histology**:	
Adenocarcinoma	25 (58.14%)
Squamous cell carcinoma	18 (41.86%)
**Asymptomatic brain metastases**:	
Yes	8 (18.60%)
No	35 (81.40%)
**PD-L1 TPS**:	
*Available*	23 (52.27%)
− 0%	4 (17.39%)
- ≥ 1% < 50%	19 (82.61%)
*Not Available*	21 (47.73%)
**Type of mAb**:	
Nivolumab	28 (63.64%)
Pembrolizumab	9 (20.45%)
Atezolizumab	7 (15.91%)
**Type of response**:	
CR	2 (4.55%)
PR	8 (18.18%)
SD	9 (20.45%)
PD	25 (56.82%)
ORR	10 (22.73%)
**Survival outcomes**:	
Median follow-up	41.46 months (12.56–53.20 months)
Median PFS	3.52 months
Median OS	8.53 months

### Genotyping and mapping of *PD-L1* SNPs in NSCLC patients treated with ICIs

rs822336, rs2282055 and rs4143815 *PD-L1* SNPs were selected and genotyped in the study population. Frequencies of the analyzed *PD-L1* SNPs are described in Table 2. rs822336 was genotyped as G/G, G/C and C/C in 11 (25.00%), 22 (50.00%) and 11 (25.00%) patients, respectively. rs2282055 was genotyped as T/T, G/T and G/G in 25 (56.82%), 15 (34.09%) and 4 (9.09%) patients, respectively. Lastly, rs4143815 was genotyped as G/G and G/C in 29 (65.91%) and 15 (34.09%) patients, respectively. No patient carried C/C genotype in rs4143815. Mapping of SNPs to *PD-L1* showed that rs822336, rs2282055 and rs4143815 were localized on its promoter/enhancer (Chr 9: 5448690), intron (Chr 9: 5455732) and 3’ untranslated regions (3’UTR) (Chr 9: 5,468,257), respectively.

**Table 2:** Frequencies of *PD-L1* SNP genotypes

Genotype	Frequency
**rs822336:**	
G/G	11 (25.00%)
G/C	22 (50.00%)
C/C	11 (25.00%)
**rs2282055**:	
T/T	25 (56.82%)
G/T	15 (34.09%)
G/G	4 (9.09%)
**rs4143815**:	
G/G	29 (65.91%)
G/C	15 (34.09%)
C/C	0 (00.00%)

### Association between clinical-pathological characteristics and clinical outcomes in NSCLC patients treated with ICIs

Age and ECOG PS of the patients significantly correlated with PFS (*P* = 0.0007 and *P* = 0.0300) and OS (*P* = 0.0008 and *P* = 0.0430). Absence of brain metastases significantly correlated with ORR (*P* = 0.05). Lastly, smoker status correlated with ORR (*P* = 0.0260), PFS (*P* = 0.0140) and OS (*P* = 0.0126). Specifically, smoker patients displayed the best ORR, PFS and OS. Among smoker patients, those who became non-smokers displayed a worse ORR, PFS and OS as compared to those who were current smokers. No significant association between irAEs or PD-L1 TPS and ORR, PFS and OS were found.

### Association between *PD-L1* SNPs and clinical-pathological characteristics or clinical outcomes in NSCLC patients treated with ICIs

**Fig. 1:**
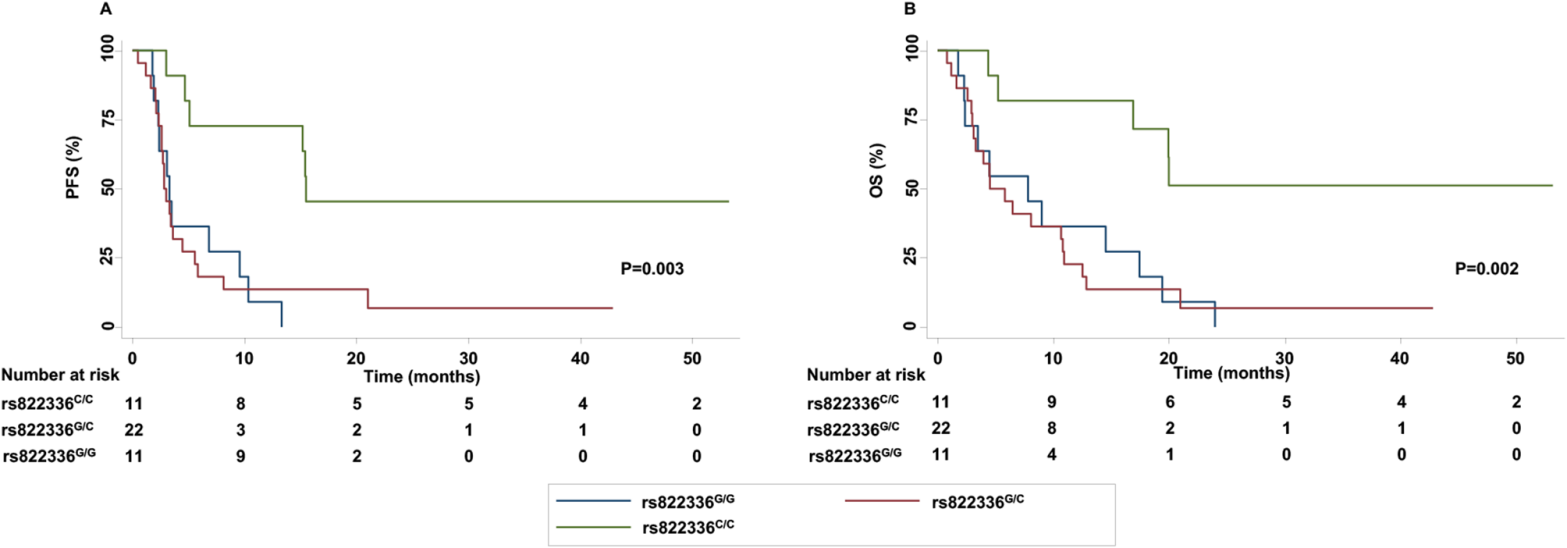
Association between rs822336 and clinical outcomes in advanced NSCLC patients treated with anti-PD-1/PD-L1 therapy. PFS (**A**) and OS (**B**) of NSCLC patients treated with anti-PD-1/PD-L1 therapy were stratified based on PD-L1 rs822336 genotypes. PFS and OS were compared using the Kaplan-Meier method. Differences in patients’ survival were analyzed using a log-rang test. *P* < 0.05 was considered statistically significant

Presence of allele G in rs822336 was associated with presence of allele G in rs2282055 (*P* = 0.000). Presence of allele C in rs822336 was associated with presence of allele T in rs2282055 (*P* = 0.000). G/C genotype in rs822336 was associated with a higher risk of hypertension as compared to homozygosis genotypes (*P* = 0.049). C/C genotype in rs4143815 was associated with PD-L1 TPS ≥ 1% (*P* = 0.033). Presence of allele C in rs822336 was associated with a lower incidence of asymptomatic brain metastases (*P* = 0.006). Specifically, patients carrying C/C or G/C genotype in rs822336 displayed a significant lower presence of asymptomatic brain metastases as compared to those carrying G/G genotype. T/T genotype in rs2282055 was slightly associated with longer PFS (*P* = 0.08) and OS (*P* = 0.09) as compared to G/T and G/G genotypes (Fig. S2). Genotype in rs4143815 did not correlate with any of clinical outcomes analyzed (Fig. S2). In contrast, patients carrying C/C genotype in rs822336 were significantly associated with better ORR (*P* = 0.004), PFS (*P* = 0.003) (Fig. 1A) and OS (*P* = 0.002) (Fig. 1B) as compared to those carrying G/C and G/G genotypes. Specifically, ORR of C/C, G/C and G/G genotypes in rs822336 were 63.63%, 13.63% and 0%, respectively. Median PFS of patients carrying C/C, G/C and G/G genotypes in rs822336 were 15.47 months (range, 3.00-53.20 months), 2.90 months (range 0.47–42.86 months) and 3.27 months (range, 1.77–13.3 months), respectively. Median OS of patients carrying C/C, G/C and G/G genotypes in rs822336 were 20.00 months (range, 4.37–53.20 months), 5.17 months (range, 0.80-42.86 months) and 7.80 months (1.77-24.00 months), respectively. Six out of 11 patients (54.54%) and 2 out of 22 patients (9.09%) carrying C/C and G/C genotypes in rs822336, respectively, were still alive at time of the analysis. No patient carrying G/G genotype in rs822336 survived. Lastly, no significant association were found between any analyzed *PD-L1* SNPs and development of irAEs. Multivariate analysis confirmed the significant association between C/C genotype in rs822336 and PFS (*P* = 0.005) (Table 3) or OS (*P* = 0.010) (Table 4).

**Table 3:** Association between C/C genotype in rs822336 and PFS of NSCLC patients treated with anti-PD-1/PD-L1 therapy: multivariate analysis

Variables	HR	*p*-value	95% CI	Univariate *p*-value
rs822336^C/C^ vs. rs822336^G/C or G/G^	0.5163762	0.005	0.3241144–0.8226861	0.0030
Current smoker vs. never /previous smoker	0.3619732	0.015	0.159869–0.8195749	0.0140
Age	0.9743548	0.254	0.9317971–1.018856	0.0007
ECOG PS 0 vs. 1/2	1.226831	0.288	0.8413328–1.788963	0.0300

**Table 4:** Association between C/C genotype in rs822336 and OS of NSCLC patients treated with anti-PD-1/PD-L1 therapy: multivariate analysis

Variables	HR	*p*-value	95% CI	Univariate *p*-value
rs822336^C/C^ vs. rs822336^G/C or G/G^	0.5424147	0.010	0.3408916–0.8630712	0.0020
Current smoker vs. never/previous smoker	0.3902317	0.016	0.1809386–0.841616	0.0126
Age	0.9963466	0.860	0.9566538–1.037686	0.0008
ECOG PS 0 vs. 1/2	1.211215	0.325	0.8269563–1.774027	0.0430

**Fig. 2:**
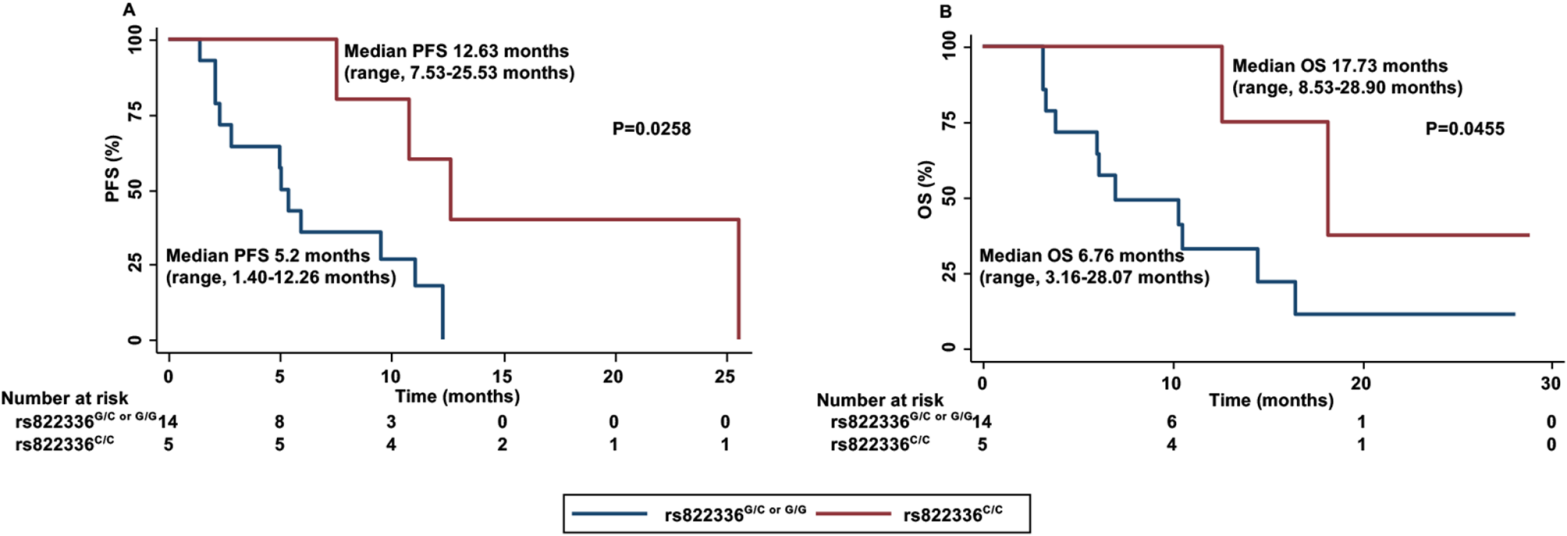
Association between rs822336 and clinical outcomes in advanced NSCLC patients treated with pembrolizumab plus platinum-based chemotherapy. PFS (**A**) and OS (**B**) of NSCLC patients treated with anti-PD-1 pembrolizumab plus platinum-based chemotherapy were stratified based on PD-L1 rs822336 genotypes. PFS and OS were compared using the Kaplan-Meier method. Differences in patients’ survival were analyzed using a log-rang test. *P* < 0.05 was considered statistically significant

To further validate these results, the predictive role of rs822336 was investigated in advanced non-oncogene addicted NSCLC patients who were treated in first line therapy with the combination of pembrolizumab plus platinum-based chemotherapy. Nineteen patients were enrolled from April 2021 to September 2023. PD-L1 TPS < 1% and 1–49% were reported in 13 (68.42%) and 6 (31.58%) of the tumors analyzed, respectively. In this patient population, C/C genotype in rs822336 was significantly associated with better PFS (*P* = 0.0258) (Fig. 2A) and OS (*P* = 0.0455) (Fig. 2B) as compared to G/C or G/G genotypes. Worth of note, PD-L1 expression was not associated either with survival outcomes (PFS (*P* = 0.8), OS (*P* = 0.4)) or with rs822336 (*P* = 0.520).

### Characterization of *PD-L1* SNPs in human NSCLC cell lines

**Fig. 3:**
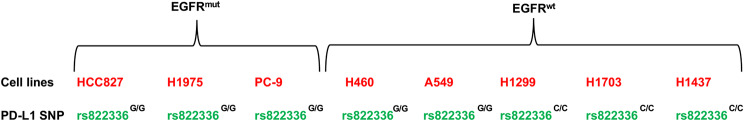
Characterization of human EGFR^mut^ and EGFR^wt^ NSCLC cell lines. HCC827, H1975, PC-9, H460, A549, H1299, H1703 and H1437 cells were seeded into 6-well plates at the density of 2 × 10^6^ per well. Following a 24 h incubation at 37 °C in a 5% CO_2_ atmosphere, cells were harvested. DNA was extracted and genotyped for rs822336 *PD-L1* SNP utilizing PCR

Promoter/enhancer region location on *PD-L1* may potentially affect gene expression changes. In addition, rs822336 efficiently predicted clinical benefit to anti-PD-1/PD-L1 mAbs in advanced non-oncogene addicted NSCLC patients. To define the mechanisms underlying the impact of rs822336 on PD-L1 expression changes as well as on anti-PD-1/PD-L1 activity, further investigation was performed in vitro. Human NSCLC cell lines carrying *EGFR* mutations (EGFR^mut^) (HCC827, H1975 and PC-9) and *EGFR* wild type (EGFR^wt^) (H460, A549, H1299, H1703 and H1437) were characterized for *PD-L1* SNPs (Fig. 3). Besides *EGFR*, H460, A549, H1299, H1703 and H1437 did not carry any alterations in *ALK, ROS1, BRAF V600, NTRK1/2*, *RET, MET* and *HER2.* EGFR^mut^ cancer cell lines were used as cancer cells expected to be resistant to anti-PD-1/PD-L1 therapy [61, 62]. EGFR^mut^ HCC827, H1975 and PC-9 cell lines as well as EGFR^wt^ H460 and A549 cells carried a G/G genotype in rs822336 (HCC827^G/G^, H1975^G/G^, PC-9^G/G^, H460^G/G^, A549^G/G^). In contrast, EGFR^wt^ H1299, H1703 and H1437 cell lines carried a C/C genotype (H1299^C/C^, H1703^C/C^, H1437^C/C^). No cell line carried a G/C genotype. To investigate any correlation between patients with rs822336 genotypes and *EGFR* mutation status in lung adenocarcinoma patients, we utilized data from TCGA, specifically selecting the Lung Adenocarcinoma Firehose Legacy dataset. Among the 586 samples, mutation data were available for 230 samples, with 33 exhibiting *EGFR* mutations. Within this subgroup, a bioinformatics analysis was conducted on Whole Genome Sequencing (WGS) data to identify the genotype of SNP rs822336. Our findings showed that 17 samples displayed the C/C genotype, 11 samples showed the G/G genotype, while WGS data were unavailable for 5 samples. Fisher’s test was applied, resulting in a no significant correlation (*P* = 0.2481) between *EGFR* status and rs822336. Further characterization of other two *PD-L1* SNPs (rs2282055 and rs4143815) (Fig. S3) in cancer cell lines validated our previously reported association in cancer patients between the allele C in rs822336 with the allele T in rs2282055.

### Association of PD-L1 expression and rs822336 in human NSCLC cell lines

**Fig. 4:**
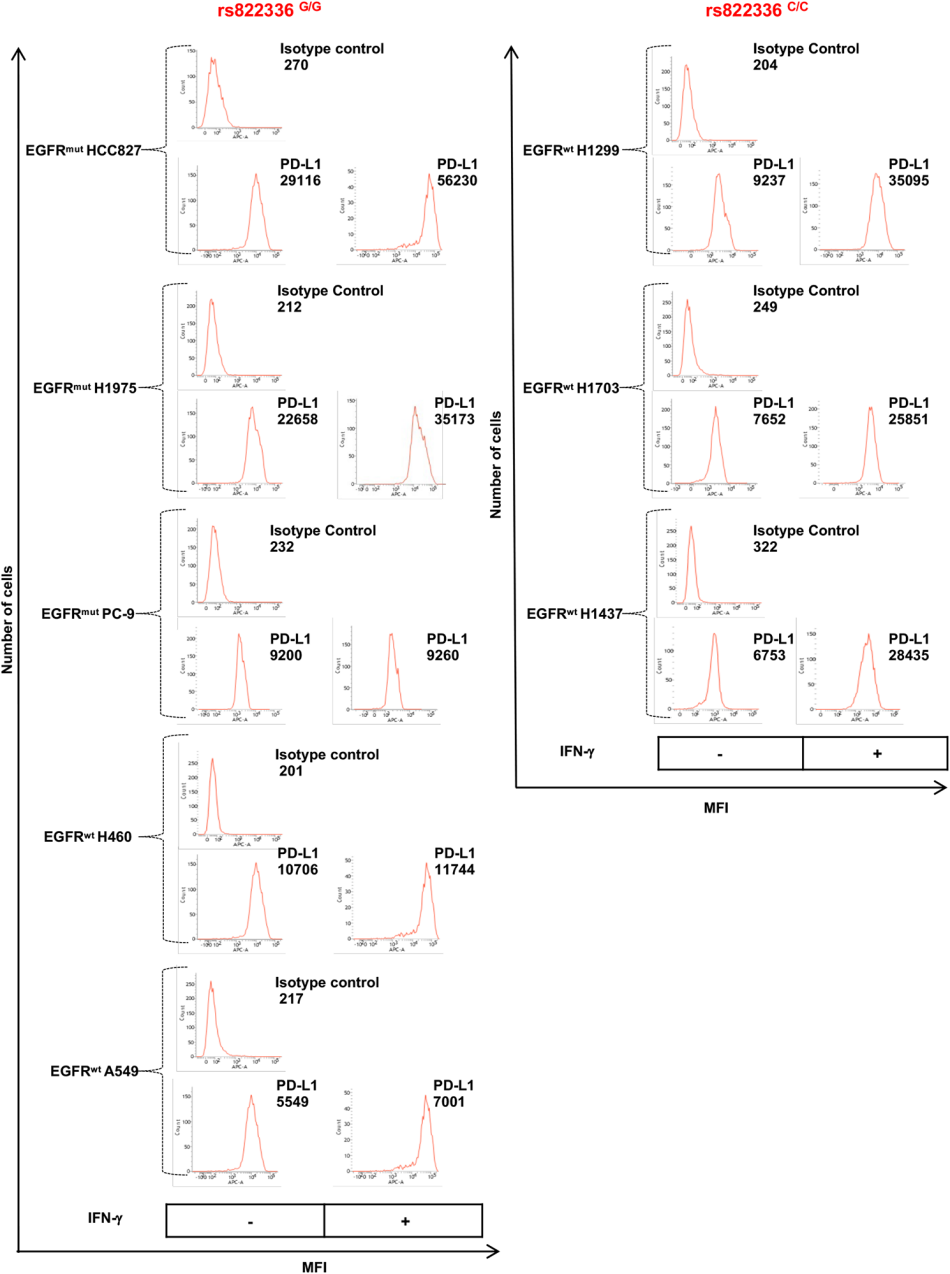
Modulation by IFN-ɣ of PD-L1 expression in NSCLC cell lines carrying different rs822336 genotype. EGFR^mut^ HCC827^G/G^, H1975^G/G^, PC-9^G/G^ and EGFR^wt^ H460^G/G^, A549^G/G^, H1299^C/C^, H1703^C/C^ and H1437^C/C^ cells were seeded into 6-well plates at a density of 2 × 10^6^ cells per well and incubated with IFN-ɣ (100ng/ml). Untreated cells were used as a control. Following a 48 h incubation at 37 °C in a 5% CO_2_ atmosphere, cells were harvested and cell surface stained with Allophycocyanin (APC) anti-PD-L1 IgG2b Ab. APC anti-mouse IgG2b was used as a specificity control. Data, expressed as mean fluorescence intensity (MFI), are representative of the results obtained in three independent experiments. No changes in MFI were observed in specificity control following IFN-ɣ incubation (data not shown)

To explore the association between rs822336 and PD-L1 expression, we determined the levels of PD-L1 mRNA and protein expression in human NSCLC cell lines carrying different rs822336 genotype, under basal conditions or following treatment with interferon gamma (IFN-ɣ) (Fig. 4 and Fig. S4). IFN-ɣ was used to induce PD-L1 up-regulation [63]. Under basal conditions, cells displayed different levels of mRNA, total protein and cell surface protein expression of PD-L1 (Fig. 4 and Fig. S4). Incubation with IFN-ɣ caused morphological changes and significantly decreased cancer cell proliferation in most of the cell lines analyzed (except in PC-9^G/G^ cells) (Fig. S5). In addition, IFN-ɣ incubation up-regulated PD-L1 expression in all cell lines, except in EGFR^mut^ PC-9^G/G^ cells. However, the extent of PD-L1 up-regulation differed based on rs822336 genotype. Specifically, following treatment with IFN-ɣ, cancer cells carrying C/C genotype in rs822336 displayed a significant higher differential increase (*P* < 0.001) in PD-L1 mRNA and protein expression as compared to cancer cells carrying G/G genotype, regardless *EGFR* status (Fig. 4 and Fig. S4).

### Modulation by rs822336 of the in vitro activity of anti-PD-1 nivolumab on NSCLC cells co-cultured with HLA class I antigen matched PBMCs

**Fig. 5:**
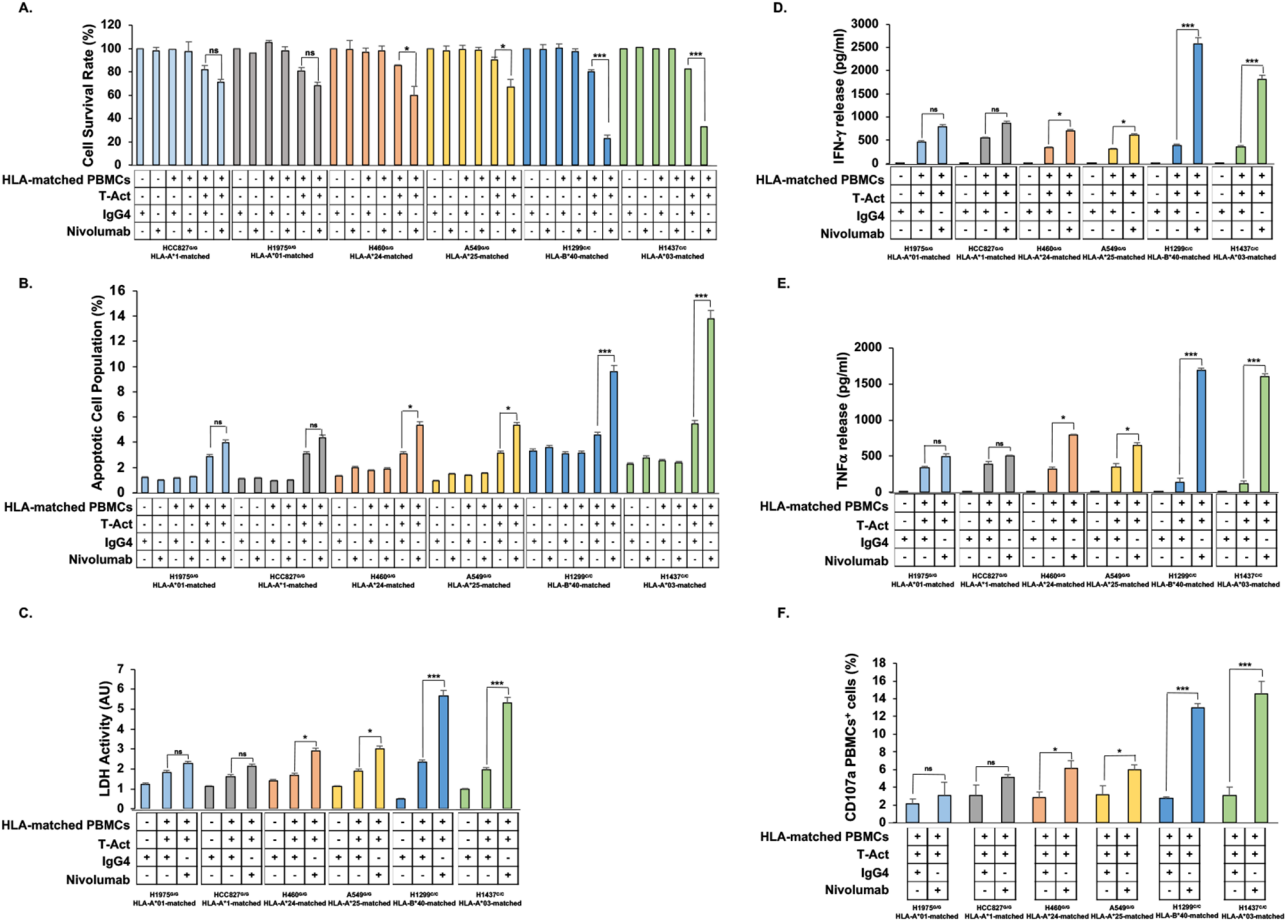
Modulation by rs822336 allele-specificity of the in vitro activity of anti-PD-1 nivolumab on NSCLC cells co-cultured with HLA-matched PBMCs. EGFR^mut^ HCC827^G/G^, H1975^G/G^ and EGFR^wt^ H460^G/G^, A549^G/G^, H1299^C/C^ and H1437^C/C^ cells were seeded into 24-well plates at a density of 2 × 10^5^ cells per well. Following a 24 h incubation at 37 °C in a 5% CO_2_ atmosphere, cells were co-cultured with HLA-matched PBMCs in a 1:10 ratio and incubated with nivolumab (10 µg/ml). HLA-A*11, HLA-A*01, HLA-A*24, HLA-A*25, HLA-B*40 and HLA-A*03 haplotypes were used for matching EGFR^mut^ HCC827^G/G^, H1975^G/G^ and EGFR^wt^ H460^G/G^, A549^G/G^, H1299^C/C^ and H1437^C/C^ cells, respectively, with PBMCs. HLA-matched PBMCs were activated utilizing an anti-CD3 (1 µg/mL) and an anti-CD28 (1 µg/mL) T Cell TransAct (T- Act). Non-activated HLA-matched PBMCs were used as controls. Purified human IgG4 was used as a control for nivolumab. (**A**) Following a 48 h incubation, cancer cell viability was determined by cell counting kit-8 (CCK-8) assay. Data are expressed as mean percentages of the viability of nivolumab treated cells ± SD as compared to cells incubated with purified human IgG4. The mean percentage of cell viability and SD were calculated from three independent experiments; each of them was performed in triplicate. (**B**) Following a 48 h incubation, apoptosis induction was determined by Annexin V/PI assay by flow cytometry. Data are expressed as mean percentages of apoptotic cell population of nivolumab treated cells ± SD as compared to cells incubated with purified human IgG4. The mean percentage of apoptotic cells and SD were calculated from three independent experiments; each of them was performed in triplicate. (**C-E)** Following a 48 h incubation, LDH (C), IFN-γ (D) and TNFα (E) levels in the medium harvested from cultures of HLA-matched PBMCs with cancer cells were measured by LDH assay kit, ELISA Max Deluxe Set Human IFN-γ kit and human TNFα ELISA, respectively. Data are expressed as means of LDH, IFN-γ and TNFα levels ± SD of the results obtained in three independent experiments; each of them performed in triplicate. (ns: not significant; *P ≤ 0.05; ***P ≤ 0.001)

To assess the functional significance of the changes induced by rs822336 on the clinical activity of anti-PD-1 based immunotherapy and/or PD-L1 induction by IFN-ɣ, we investigated the effect of anti-PD-1 mAb nivolumab on the recognition of NSCLC cells carrying different rs822336 genotypes by activated HLA class I antigen matched peripheral blood mononuclear cells (HLA-matched PBMCs). Human NSCLC cell lines were first characterized for HLA class I haplotype profiles as well as expression of HLA class I/II antigens and β2m (Fig. S6). All cell lines did not show any significant alteration of HLA class I, HLA class II antigen and β2m expression. HLA haplotype of each cell lines is shown in Fig. S6. EGFR^mut^ HCC827^G/G^, H1975^G/G^ and EGFR^wt^ H460^G/G^, A549^G/G^, H1299^C/C^ and H1437^C/C^ cells were selected for further experiments based on their common histology (adenocarcinoma) and their different status of EGFR and rs822336. HLA-A*11, HLA-A*01, HLA-A*24, HLA-A*25, HLA-B*40 and HLA-A*03 haplotypes were used for matching EGFR^mut^ HCC827^G/G^, EGFR^mut^ H1975^G/G^, EGFR^wt^ H460^G/G^, EGFR^wt^ A549^G/G^, EGFR^wt^ H1299^C/C^ and EGFR^wt^ H1437^C/C^ cells, respectively, with PBMCs. In all cell lines co-culturing of activated HLA-matched PBMCs with cancer cells significantly (*P* < 0.001) decreased cancer cell viability (Fig. 5A) and increased apoptotic cancer cells (Fig. 5B), PBMC-mediated LDH (Fig. 5C), IFN-γ (Fig. 5D), TNFα (Fig. 5E) releases as well as the percentage of positive CD107a PBMCs (Fig. 5F) as compared to appropriate control. Treatment with nivolumab of all cell lines did not influence cancer cell viability and PBMC activation as well as cancer cell apoptosis induction as compared to cells treated with isotype control, even when cancer cells were incubated with not activated HLA-matched PBMCs. However, treatment with nivolumab significantly (*P* < 0.001) and differentially affected the activated PBMC-mediated effects based on rs822336 genotype. Specifically, in cancer cells carrying C/C genotype treatment with nivolumab dramatically decreased cancer cell survival (Fig. 5A) and increased apoptotic cancer cells (Fig. 5B), PBMC-mediated LDH (Fig. 5C), IFN-γ (Fig. 5D), TNFα (Fig. 5E) releases as well as the percentage of positive CD107a PBMCs (Fig. 5F) as compared to cells treated with isotype control and co-cultured with activated matched PBMCs. In contrast, in a significant less extent PBMC activation as well as cancer cell death were detected when EGFR^mut^ or EGFR^wt^ cancer cells carrying G/G genotype were co-cultured with activated matched PBMCs and treated with nivolumab as compared to cells treated with isotype control and co-cultured with activated matched PBMCs. This data validated the role of rs822336 on in vitro sensitivity of NSCLC cells to anti-PD-1.

### Identification of TFs binding to rs822336 based on its allele-specificity

**Fig. 6:**
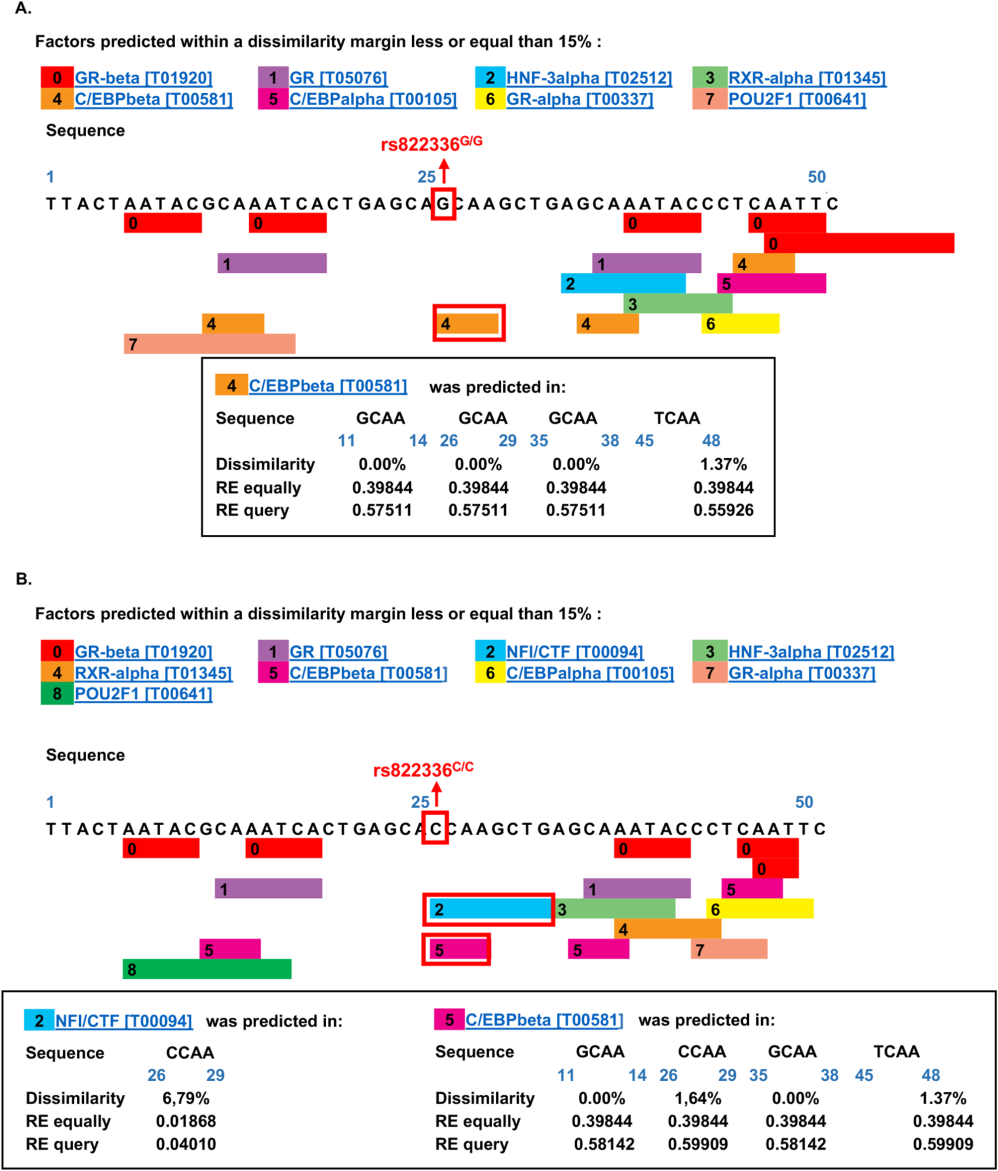
Prediction of TF binding to rs822336 based on allele-specificity. The output of the analysis contains the TFs [database accession number] predicted with a dissimilarity margin less or equal than 15% to the target sequence +/−25 bp to rs822336 G (**A**) and C (**B**) genotype. Nucleotide sequence of the potential binding site as well as the position of each TFBS are illustrated. Tables report a detail of the serum response factor (SRF) binding site prediction and the random expectation (RE) values for different levels of sequence-matrix similarity. The rate of dissimilarity between the putative and consensus sequences for TFs is indicated; RE indicates expected occurrences of the match in a random sequence of the same length as the query sequence according to the dissimilarity index based on equi–probability for the four nucleotides (RE equally) and nucleotide frequencies in the query sequence (RE query). NFI/CTF [T00094] also known as Nuclear Factor I C (NFIC)

SNPs localized in TFBSs can affect TF binding affinity that in turn modifies target gene expression. rs822336 mapped to the promoter/enhancer region of *PD-L1.* In order to study the mechanisms underlying the modulation by rs822336 allele-specificity of the in vitro activity of anti-PD-1 associated to a differential induction of PD-L1 expression on NSCLC cells we investigated whether differential TFs binding to rs822336 allele-specificity might affect *PD-L1* expression. The high-quality transcription factor binding profile databases Promo 3.0, Expasy, Transfac and Jaspar were used. As reported in Fig. 6, CCAAT-enhancer-binding protein beta (C/EBPβ) and Nuclear Factor I C (NFIC) TFs were identified to uniquely bind to rs822336 based on its allele-specificity. Specifically, both C/EBPβ and NFIC bound to rs822336 in presence of C/C genotype (dissimilarity rate by Promo 3.0: 0.0% and 6.79% for C/EBPβ and NFIC, respectively) while C/EBPβ alone exclusively bound to rs822336 in presence of G/G genotype (dissimilarity rate by Promo 3.0: 0.0% for C/EBPβ).

### Validation of C/EBPβ and NFIC binding to rs822336 based on its allele-specificity

**Fig. 7:**
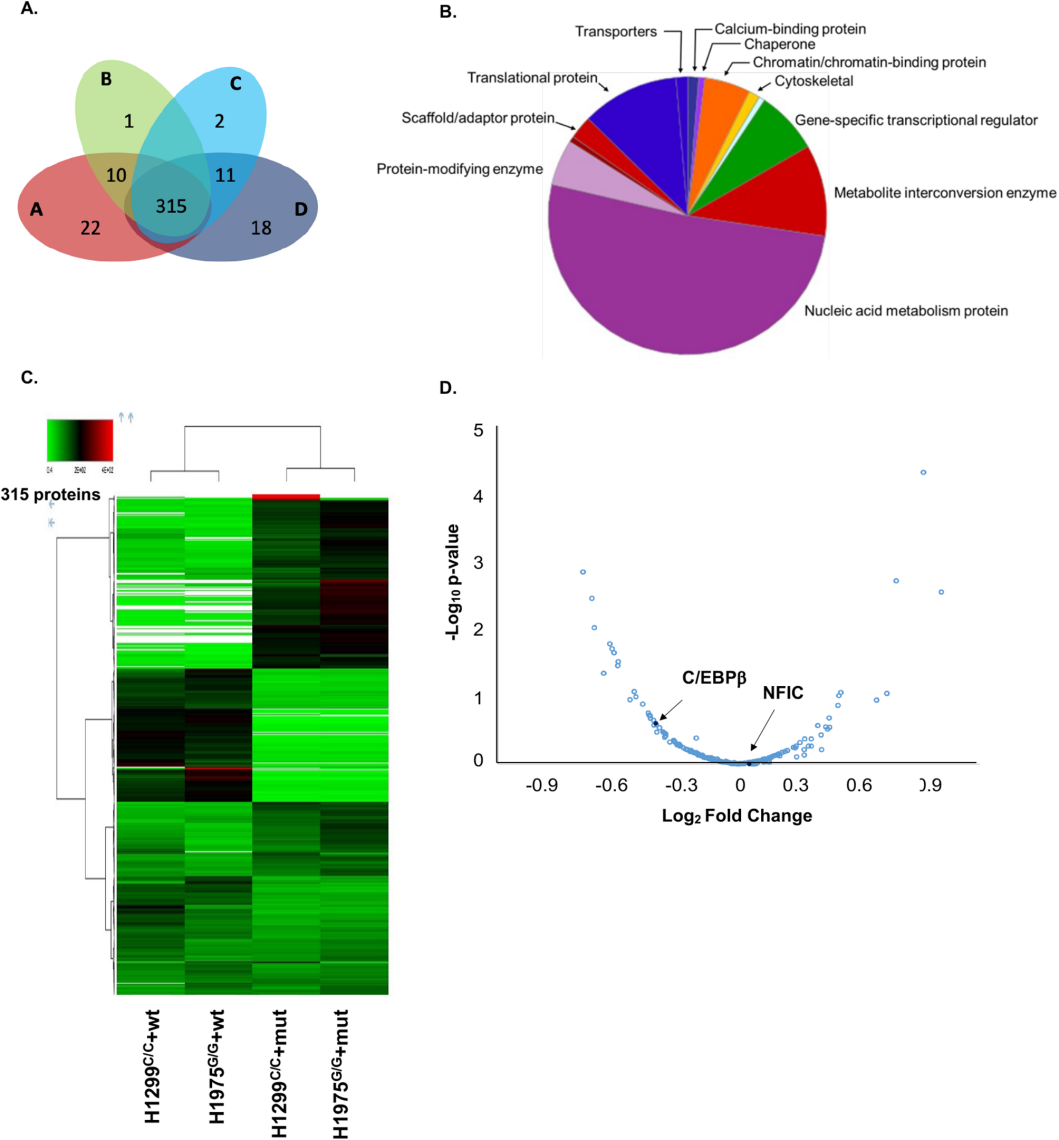
Validation of TFs involved in the regulation of *PD-L1* based on rs822336 allele-specificity. H1975^G/G^ and H1299^C/C^ cells were seeded into T75 flasks at a density of 5 × 10^6^ cells. Following a 48 h incubation at 37 °C in a 5% CO_2_ atmosphere, DNA-pull down assay was performed with immobilized wt/mut oligos incubated with nuclear extracts on 4 distinct sample group combinations. Nuclear extract of H1299^C/C^ cells was incubated with the mut oligo; nuclear extract of H1299^C/C^ cells was incubated with the wt oligo; nuclear extract of H1975^G/G^ cells was incubated with the mut oligo; nuclear extract of H1975^G/G^ cells was incubated with wt oligo. Putative TFs of the rs822336 region of *PD-L1* based on its allele-specificity were detected by LC-MS/MS. (**A**) Venn diagram represents the overlap of 315 TFs detected over the 4 analysed sample groups. (**B**) Pie chart shows the gene ontology (GO) classification of TFs detected over the 4 analysed sample groups. (**C**) Heat map summarises the average amount measured based on the observed label-free quantitation (LFQ) intensities for each of the 315 proteins detected in the 4 analysed sample groups. (**D**) Volcano plot shows a proteomic based comparison between H1299^C/C^+mut and H1975^G/G^+wt. Reported points indicate proteins that display both large magnitude fold-changes (x axis) and high statistical significance (y axis). Corresponding points to C/EBPβ and NFIC are highlighted

We then validated the role of rs822336 genotype in modulating a competitive affinity binding of NFIC and C/EBPβ to the TFBS of *PD-L1* promoter/enhancer region. H1975^G/G^ and H1299^C/C^ were used to perform the DNA-protein pull-down based proteomic approach using double-stranded oligos immobilized on biotinylated spheres, carrying either the G (wild type) or C (mutant) genotype on rs822336. Comparison of nuclear extracts of H1975^G/G^ or H1299^C/C^ cells with obtained immobilized spheres identified 315 common proteins as putative interactors of rs822336 region of *PD-L1* (Fig. 7A). More than 70% of these proteins were classified as those involved in cell metabolism and DNA/RNA binding proteins (Fig. 7B). The latter included 11 gene-specific TFs (Table 5). In addition, comparison of the relative abundance of the total common proteins identified from the corresponding nuclear extracts of H1299^C/C^ and H1975^G/G^ cell lines with mut (H1299^C/C^+mut and H1975^G/G^+mut) or wt oligo (H1299^C/C^+wt and H1975^G/G^+wt) revealed significant difference based on interacting oligos rather than nuclear extract of the cell lines (Fig. 7C). Specifically, samples obtained from the interaction of the same oligo with different nuclear extract obtained from each cell line displayed high levels of similarity in protein distribution (H1299^C/C^+wt vs. H1975^G/G^+wt; H1299^C/C^+mut vs. H1975^G/G^+mut). In contrast, samples obtained from the interaction of different oligos with nuclear extracts obtained from each cell line displayed high levels of difference in protein distribution (H1299^C/C^+mut vs. H1299^C/C^+wt; H1975^G/G^+mut vs. H1975^G/G^+wt). The two pairs of samples obtained with same oligo with two different nuclear extracts differed from the other two since a different third of the proteins in each pair of samples showed a greater affinity based on the corresponding oligo. Focusing on two physiological conditions, the amount of C/EBPβ but not of NFIC (abundance ratio 1.09) significantly differed in the nuclear extract of H1299^C/C^ cells with mut oligo (H1299^C/C^+mut) as compared to that of H1975^G/G^ cells with wt oligo (H1975^G/G^+wt) (H1299^C/C^+mut / H1975^G/G^+wt abundance ratio: 0.32 C/EBPβ, 1.09 NFIC) (Fig. 7D and Fig S7) (Supplementary file 2).

**Table 5:** Gene-specific transcription factors (TFs) identified by DNA pull-down assay

GENE ID	GENE
P08651	Nuclear factor 1 C-type (NFIC)
P17676	CCAAT/enhancer-binding protein beta (CEBPB)
Q00577	Transcriptional activator protein Pur-alpha (PURA)
Q03701	CCAAT/enhancer-binding protein zeta (CEBPZ)
Q15650	Activating signal cointegrator 1 (TRIP4)
Q16666	Gamma-interferon-inducible protein 16 (IFI16)
Q8WU90	Zinc finger CCCH domain-containing protein 15
Q96HQ2	CDKN2AIP N-terminal-like protein (CDKN2AIPNL)
Q96NXV6	CDKN2A-Interacting protein (CDKN2AIP)
Q96QR8	Transcriptional activator protein Pur-beta (PURB)
Q9H147	Deoxynucleotidyltransferase terminal-interacting protein 1 (DNTTIP1)

### Characterization of the mechanisms underlying differential induction of PD-L1 expression by C/EBPβ and NFIC based on rs822336 allele-specificity

**Fig. 8:**
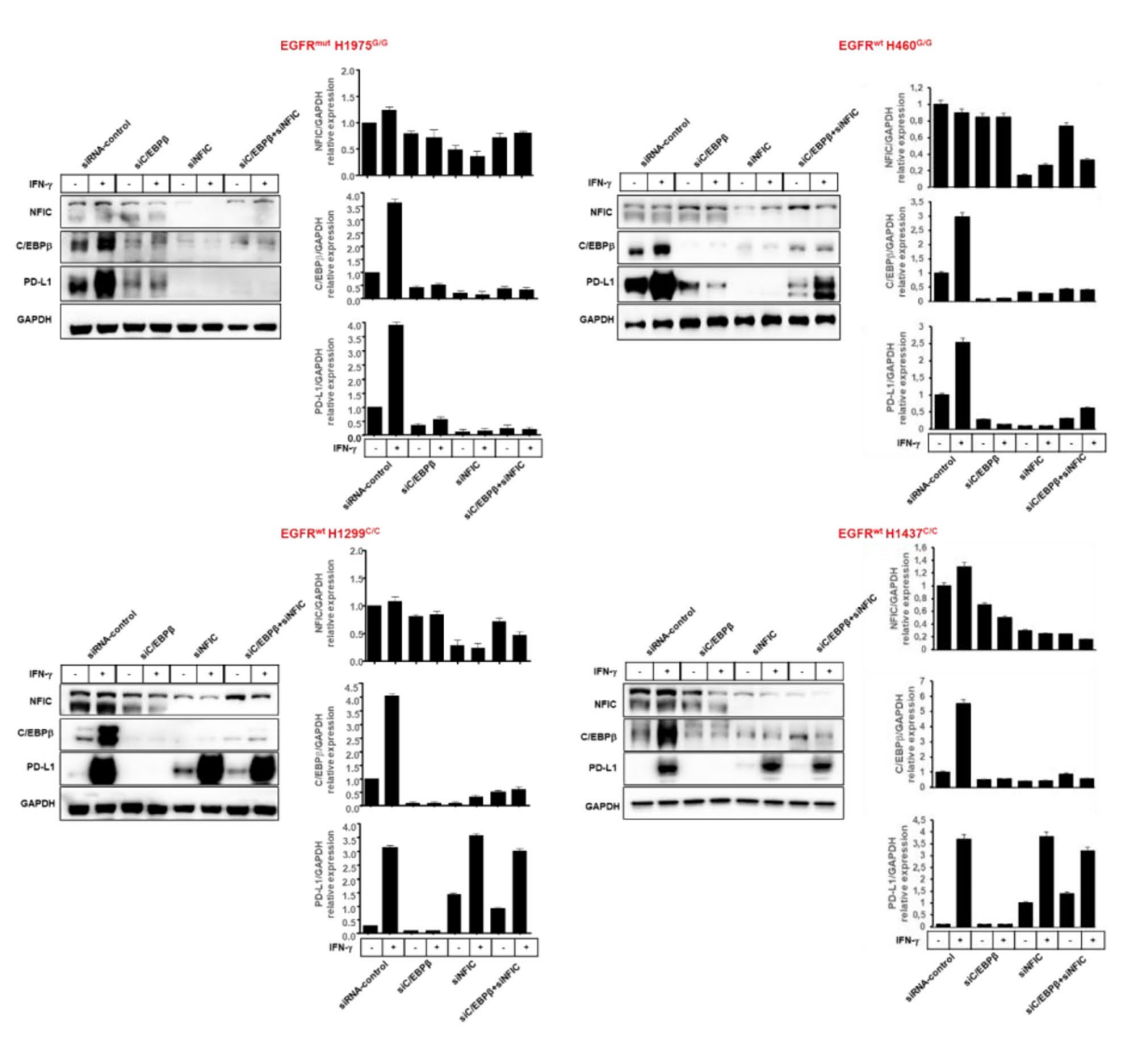
Regulation by C/EBPβ and NFIC silencing of PD-L1 expression in NSCLC cell lines carrying different rs822336 genotype under basal conditions or following IFN-ɣ incubation. EGFR^mut^ H1975^G/G^ and EGFR^wt^ H460^G/G^ cells (upper panel) as well as EGFR^wt^ H1299^C/C^ and EGFR^wt^ H1437^C/C^ cells (lower panel), transduced with C/EBPβ- and NFIC-specific siRNAs or siRNA-controls, were seeded into 6-well plates at a density of 2 × 10^6^ cells per well and incubated with IFN-ɣ (100ng/ml). Untreated cells were used as a control. Following a 48 h incubation at 37 °C in a 5% CO_2_ atmosphere, cells were harvested and lysed. Cell lysates were analyzed by western blot with NFIC, C/EBPβ and PD-L1-specific Abs. Representative results are shown. The levels of NFIC and C/EBPβ, normalized to GAPDH and relative to untreated siRNA-control as well as levels of PD-L1, normalized to GAPDH and relative to treated siRNA-control, are plotted on the right, and expressed as mean ± SD of the results obtained in three independent experiments

Three small interfering RNAs (siRNAs) were used to silence C/EBPβ (SR300760A, SR300760B, SR300760C) or NFIC (SR303158A, SR303158B, SR303158C) in EGFR^mut^ H1975^G/G^ and EGFR^wt^ H1299^C/C^ cell lines. SR300760B and SR303158B, respectively, were selected for further silencing of C/EBPβ and NFIC (Fig. S8). To study the regulation by C/EBPβ and NFIC silencing of PD-L1 expression, EGFR^mut^ H1975^G/G^ and EGFR^wt^ H460^G/G^, H1299^C/C^ and H1437 ^C/C^ cells were transduced under basal conditions or following IFN-ɣ incubation. As shown in Fig. 8 and Fig. S9, C/EBPβ and NFIC silencing markedly and differentially changed both PD-L1 protein and mRNA expression in all cell lines. Specifically, in EGFR^wt^ H1299^C/C^ and H1437^C/C^ cells, expected to bind both C/EBPβ and NFIC on rs822336, C/EBPβ and NFIC silencing significantly decreased and increased PD-L1 levels, respectively. In contrast, in EGFR^mut^ H1975^G/G^ and EGFR^wt^ H460^G/G^ cells, expected to bind C/EBPβ alone on rs822336, PD-L1 levels were significantly decreased by C/EBPβ silencing but were not increased by NFIC silencing. In line with previous experiments, incubation with IFN-ɣ of cancer cells more markedly increased the expression levels of PD-L1 in EGFR^wt^ H1299^C/C^ and H1437^C/C^ cells as compared to EGFR^mut^ H1975^G/G^ and EGFR^wt^ H460^G/G^ cells. This effect was associated with a marked increase of C/EBPβ. No changes in NFIC in all cell lines were detected following IFN-ɣ incubation. Increased levels of STAT1 phosphorylation at Ser-727 in cancer cells incubated with IFN-ɣ indicated IFN-ɣ pathway activation [64] (Fig. S10). However, a differential effect on IFN-ɣ-mediated PD-L1 up-regulation based on allele-specificity of rs822336 was detected in cancer cells silenced for C/EBPβ and/or NFIC (Fig. 8 and Fig. S9). Specifically, in all cell lines, IFN-ɣ incubation did not overcome C/EBPβ silencing-mediated PD-L1 decrease. In contrast, in EGFR^wt^ H1299^C/C^ and H1437^C/C^ cells but not in EGFR^mut^ H1975^G/G^ and EGFR^wt^ H460^G/G^ cells, NFIC silencing more markedly increased IFN-ɣ-mediated PD-L1 up-regulation. Lastly, in EGFR^mut^ H1975^G/G^ and EGFR^wt^ H460^G/G^ cells, the concurrent application of C/EBPβ-specific and NFIC-specific siRNAs underscored the heightened influence of C/EBPβ activity on PD-L1 expression when compared to NFIC, in both baseline conditions and post-IFN-ɣ incubation. Conversely, in EGFR^wt^ H1299^C/C^ and H1437^C/C^ cells, dual targeting emphasized the more pronounced impact of NFIC activity on PD-L1 expression in contrast to C/EBPβ. These results validated the influence of C/EBPβ and NFIC on both expression and IFN-ɣ mediated induction of PD-L1 in presence of C/C genotype as well as the lack of influence of NFIC on PD-L1 in presence of G/G genotype. In addition, these results validated C/EBPβ and NFIC as activator and repressor, respectively, of *PD-L1* based on rs822336 allele-specificity, regardless of IFN-ɣ pathway activation. Worth of note, NFIC silencing decreased CEBPβ expression in both cell lines even in presence of IFN-ɣ as compared to siRNA control. Analysis of ENCODE Transcription Factor Dataset demonstrated that C/EBPβ is a target of NFIC.

## Discussion

Anti-PD-1/PD-L1 therapy has revolutionized the therapeutic management of several types of cancer including NSCLC [16–22, 65]. This novel therapy has been shown to improve the survival of patients with advanced non-oncogene addicted NSCLC as compared to standard chemotherapy [18–20, 22, 24–27]. Unfortunately, only 13–16% of patients achieve long term benefit from this therapy and there is still the need to identify efficient predictive biomarkers of treatment response. PD-L1 tumor expression has been widely explored as a predictive biomarker. Several studies have shown a significant association between PD-L1 tumor expression and an increased likelihood of tumor response [26, 27, 30, 32]. Nevertheless, many others have also shown that patients who do not express PD-L1 in the tumor microenvironment may also benefit from anti-PD-1/PD-L1-based immunotherapy [29, 30, 32, 33]. In addition, clinical response to PD-1 blockade has been also linked to IFN-γ–related mRNA profile. The latter utilized a T cell inflamed GEPs as a marker of IFN-γ–induced genes including PD-L1 [66]. However, even in this case anti-PD-1/PD-L1 clinical benefits are not efficiently predicted. Overall, PD-L1 tumor expression is considered an inefficient biomarker, currently used as a “surrogate” for predicting cancer patients who are more likely to benefit from anti-PD-1/PD-L1 therapy [29, 30, 32, 33, 67].

In the past few years, *PD-L1* SNPs have been investigated for their potential role as predictive biomarkers of tumor response to anti-PD-1/PD-L1 therapy with contrasting results [52, 68]. In the present work, we show that not all *PD-L1* SNPs have clinical significance for anti-PD-1/PD-L1 therapy since only rs822336 significantly correlates with treatment response and survival outcomes. Specifically, C/C genotype in rs822336 is associated with better ORR, PFS and OS as compared to G/C and G/G genotypes, identifying patients long surviving. In contrast, all and most of patients carrying GG and GC genotypes, respectively, are not survived. Worth of note, PD-L1 tumor expression does not correlate with rs822336 as well as with survival outcomes. As a result, even in our case PD-L1 expression (TPS) does not identify patients long surviving.

Currently, advanced non-oncogene addicted NSCLC patients are treated in first line either with the combination of anti-PD-1/PD-L1 mAbs and chemotherapy or with anti-PD-1/PD-L1 mAbs alone, based on PD-L1 tumor expression < 50% or ≥ 50% [26, 27]. As a result, those with a PD-L1 tumor expression < 50% are most likely to display less benefit from immunotherapy alone. Our patient populations included non-oncogene addicted NSCLC patients carrying tumors with a PD-L1 expression of less than 50%, who were treated with either immunotherapy alone as second line or anti-PD-1 pembrolizumab and platinum-based chemotherapy in front line. In these patients, prediction by rs822336 identifies a high percentage of patients long surviving.

rs822336 has already been reported to display a prognostic role for NSCLC patients. For instance, a significant correlation between C/C genotype and OS in NSCLC patients is reported by Zhao et al. as well as by Kang et al. [69, 70]. In contrast, no significant difference in survival outcomes based on rs822336 genotype is reported [48, 71, 72]. Here, we do not analyze the prognostic role of *PD-L1* SNPs. We show for the first time that rs822336 in *PD-L1* efficiently predicts long term clinical benefit from ICI-based immunotherapy in advanced non-oncogene addicted NSCLC patients. The representativeness of the patient populations included in our analysis is validated by the percentage of long surviving patients following treatment with anti-PD-1/PD-L1 therapy, being in line with data reported in the literature [73–75]. Besides rs822336, other two *PD-L1* SNPs (rs4143815 and rs2282055) were included in our analysis. All three SNPs were selected based on their reported role in autoimmunity, cancer predisposition and cancer prognosis [48–50]. However, to the best of our knowledge, no clear evidence about the predictive role as well as the functional significance of these *PD-L1* SNPs is available in the literature. In the present work, a C/C genotype in rs4143815 significantly correlates with PD-L1 tumor expression (TPS ≥ 1%) but not with OS or PFS. Similar results were also obtained by M.-K. Yeo et al. [71]. In contrast, both C/C and G/C genotypes in rs4143815 were reported by Nomizo et al. to be significantly associated with better ORR and PFS as compared to G/G genotype. In the same work, presence of allele G in rs2282055 correlated with better ORR and PFS as compared to allele T. In the present study, T/T genotype in rs2282055 is slightly correlated with longer PFS and OS as compared to G/T and G/G genotypes, not reaching statistical significance. Many reasons might underlie these differences. First, the clinical-pathological characteristics of the study population as well as the allele frequencies of analyzed *PD-L1* SNPs differ between the studies. Comparison of allele frequency distribution of *PD-L1* SNP with that of Caucasian (Italian) patients obtained from 1000 Genomes Project (https://www.ensembl.org) analysis corroborated the validity of our patient population. Second, we find a significant association between presence of allele C in rs822336 and that of allele T in rs2282055. As a result, the slight clinical significance of allele T in rs2282055 might reflect the statistical clinical significance of allele C in rs822336. Lastly, mapping of *PD-L1* SNPs shows that rs822336 and rs2282055 are localized on *PD-L1* promoter/enhancer and intron region, respectively. Promoter/enhancer regions, more than intron regions, play a crucial role in gene expression since they represent a binding site for TFs involved in gene expression regulation [37–39]. As a result, rs822336 is expected to affect TF binding affinity that in turn can modify target gene expression [37–39]. To validate this hypothesis, we demonstrate for the first time that rs822336 modulates PD-L1 expression and even more importantly that induction of PD-L1 expression more than its basal levels is associated to an increased sensitivity of cancer cells to immune cells. Specifically, we show in vitro that basal levels of PD-L1 in NSCLC cells carrying C/C genotype in rs822336 are lower as compared to those of G/G genotype including EGFR^mut^ cells. The latter expressing G/G genotype have been used as cancer cells expected to be resistant to anti-PD-1/PD-L1 therapy. Indeed, oncogene addicted NSCLC patients such as those carrying *EGFR* alterations are shown to be resistant to anti-PD-1/PD-L1 mAbs [76] although there is also data suggesting that activating *EGFR* mutations led to PD-L1 upregulation in NSCLC as well as animal models showing increase survival after PD-1 therapy in EGFR driven adenocarcinoma [77–80]. In our case all cancer cells, including EGFR^mut^ and EGFR^wt^ cells, carrying a G/G genotype in rs822336 expressed higher basal levels of PD-L1 as compared to cells carrying a C/C genotype. However, treatment with IFN-ɣ induced a significant higher differential increase in PD-L1 expression only in cancer cells carrying C/C genotype as compared to those with G/G genotype, including both EGFR^mut^ and EGFR^wt^ cells. IFN-ɣ is utilized to mimic the state of the tumor microenvironment as well as to induce PD-L1 expression [63]. The correlation between PD-L1-mRNA expression and rs822336 genotype has already been reported by Zhao et al. in PBMC obtained from cancer patients carrying different rs822336 genotype [70]. However, to the best of our knowledge, no study has shown that a differential induction of PD-L1 expression in NSCLC cells is influenced by *PD-L1* SNPs. Whether this represents a general phenomenon should be further investigated. It is worth of note that we also investigated whether a correlation exists between rs822336 genotype and G/G status in EGFR^mut^ NSCLC. Our data shows that no significant correlation exists. However, we did not investigate whether a C/C genotype in rs822336 in EGFR^mut^ cell line is also associated with an increased induction of PD-L1 by IFN-ɣ. As a result, further investigation in this type of cancer cells is required.

To study the mechanisms underlying the clinical significance of rs822336 genotypes on the induction of *PD-L1* more than on its basal level of expression, we first identify C/EBPβ and NFIC as the TFs of *PD-L1* promoter/enhancer region whose binding site is affected by allele-specificity of rs822336. Specifically, we show that in presence of allele C in rs822336, *PD-L1* transcription is regulated by the binding of both C/EBPβ and NFIC. In contrast, only C/EBPβ regulates *PD-L1* transcription in presence of allele G. C/EBPβ is a TF that promotes the expression of many genes involved in several cellular processes such as apoptosis, cell differentiation, inflammation and tumorigenesis [81, 82]. In contrast, NFIC is a TF involved in chromatin remodeling and epigenetic modulation by acting as activator or repressor of gene expression [83]. Consistent with these results, our data demonstrates that C/EBPβ and NFIC play a major role as activator and repressor, respectively, of PD-L1 expression based on allele-specificity of rs822336 genotype. Specifically, silencing of C/EBPβ, even in presence of IFN-ɣ incubation, decreases the levels of PD-L1 in both NSCLC cells carrying different rs822336 genotypes, regardless of EGFR status. In contrast, silencing of NFIC increases the levels of PD-L1 in NSCLC cells carrying C/C genotype but not in those with G/G genotype, including EGFR^mut^ and EGFR^wt^ cells. In the latter cells, the lack of PD-L1 regulation by NFIC silencing is likely to reflect the inability of NFIC to bind the allele G in rs822336. To validate these results, we demonstrate that among all common proteins binding *PD-L1* promoter/enhancer region on rs822336, there is a high abundance of C/EBPβ alone and not of NFIC in presence of G/G genotype. In contrast, both C/EBPβ and NFIC are found in presence of C/C genotype. It is worth of note that in presence of IFN-ɣ incubation induction of PD-L1 expression is associated to a significant high increase of C/EBPβ but not of NFIC, regardless of rs822336 genotype. As a result, in cancer cells carrying C/C genotype the significant higher differential increase in PD-L1 induction is likely to reflect the unbalanced competitive binding induced by IFN-ɣ on C/EBPβ and NFIC. The latter function as activator and repressor, respectively, of *PD-L1*. In contrast in cancer cells carrying G/G genotype the lack of a marked PD-L1 induction is likely to reflect the already high levels of PD-L1 because of binding of C/EBPβ alone that functions as activator.

**Fig. 9:**
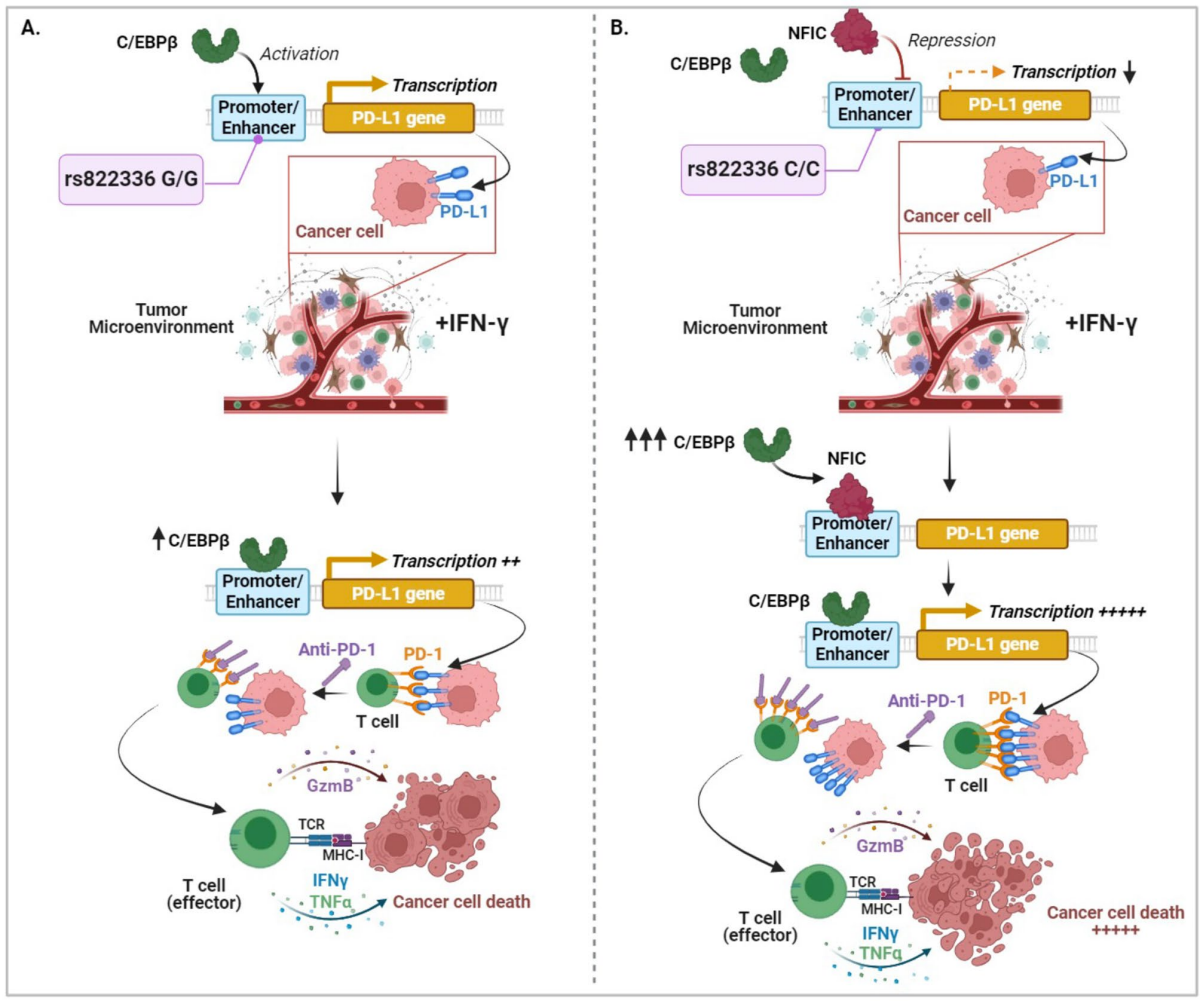
Graphical representation of the influence of rs822336 genotype on PD-L1 induction and increased sensitivity of NSCLC cells to anti-PD-1/PD-L1 based therapy

Lastly, the functional significance of the changes induced by rs822336 on the induction of PD-L1 expression as well as the predictive value of rs822336 on the sensitivity to anti-PD-1/PD-L1-based immunotherapy is demonstrated by the in vitro differential recognition of NSCLC cells carrying different rs822336 genotypes utilizing HLA-matched PBMCs, and incubation with anti-PD-1 nivolumab. PBMCs were obtained from HLA-matched healthy donors to reduce the HLA unmatched-cytotoxic PBMC-mediated effects on co-cultured NSCLC cells [84]. Studies of the integrity of HLA class I and II expression as well as of β2m expression in analyzed NSCLC cell lines excluded other potential mechanisms of immune escape. Nivolumab slightly increased the PBMC recognition and destruction of NSCLC cells carrying G/G genotype in both EGFR^mut^ and EGFR^wt^ cells. These cells were expected to have a low increase of IFN-ɣ-mediated induction of PD-L1 expression because of binding of C/EBPβ alone to the promoter/enhancer region of *PD-L1* which in turn causes an already high PD-L1 basal expression. In contrast, nivolumab dramatically increases the PBMC recognition and destruction of NSCLC cells carrying C/C genotype, markedly increasing IFN-ɣ-mediated PD-L1 expression because of modulation of the competitive binding of both C/EBPβ and NFIC to *PD-L1* promoter/enhancer region (Fig. 9). We did not investigate whether in EGFR^mut^ cells carrying C/C genotypes there is also an increased susceptibility to anti-PD-1/PD-L1 therapy. Several mechanisms can impair the susceptibility of EGFR^mut^ cells to anti-PD-1/PD-L1 therapy such as a low tumor mutation burden as well as CD73/adenosine pathway activation [85, 86]. As a result, whether rs822336 genotype can also influence the susceptibility to anti-PD-1/PD-L1 therapy in EGFR^mut^ NSCLC should be further investigated.

This study presents some limitations such as the small sample size of the study populations, the limited availability of PD-L1 TPS and the partial characterization of specific actionable oncogene alterations in all tumor samples. TPS evaluation as well as oncogene alterations were obtained from available pathological data obtained from patients treated without interfering with clinical practice. However, confirmation of major prognostic factors such as age, ECOG PS, absence of asymptomatic brain metastases and smoking status further supports the validity of the study population included. Lastly, in our study, we do not investigate the potential mechanisms underlying the clinical significance of rs4143815 and rs2282055. Further studies are needed to investigate their potential role in anti-PD-L1-mediated immune response.

## Conclusion

Overall, we believe that our findings have high clinical relevance since identify rs822336 and induction of PD-L1 expression as novel and efficient predictive biomarkers of tumor response to anti-PD-1/PD-L1 therapy in patients with advanced non-oncogene addicted NSCLC.

### Electronic supplementary material

Below is the link to the electronic supplementary material.

Additional file 1: **supplementary file 1** Sequences of double-stranded oligos utilized (biotinylation at the 5′-end).

Additional file 2: **figure S1** PFS and OS of advanced NSCLC patients treated with anti-PD-1/PD-L1 therapy. At a median follow-up of 41.46 months (range, 12.56–53.20 months) median PFS and OS were 3.52 months (**A**) and 8.53 months (**B**), respectively. PFS and OS analysis was performed using the Kaplan-Meier method.

Additional file 3: **figure S2** Association between rs2282055 or rs4143815 and clinical outcomes in advanced NSCLC patients treated with anti-PD-1/PD-L1 therapy. PFS (**A**) and OS (**B**) of NSCLC patients treated with anti-PD-1/PD-L1 therapy were stratified based on PD-L1 rs2282055 (**upper panel**) or rs4143815 (**bottom panel**) genotypes. PFS and OS were compared using the Kaplan-Meier method. Differences in patients’ survival were analyzed using a log-rang test. *P* < 0.05 was considered statistically significant.

Additional file 4: **figure S3** Characterization of human EGFR^mut^ and EGFR^wt^ NSCLC cell lines. HCC827, H1975, PC-9, H1299, H1703 and H1437 cells were seeded into 6-well plates at the density of 2 × 10^6^ per well. Following a 24 h incubation at 37 °C in a 5% CO_2_ atmosphere, cells were harvested. DNA was extracted and genotyped for rs2282055 or rs4143815 *PD-L1* SNPs utilizing PCR.

Additional file 5: **figure S4** Modulation by IFN-ɣ of PD-L1 expression in NSCLC cell lines carrying different rs822336 genotype. (**A**) EGFR^mut^ HCC827^G/G^, H1975^G/G^, PC-9^G/G^ and EGFR^wt^ H1299^C/C^, H1703^C/C^ and H1437^C/C^ cells were seeded into 24-well plates at a density of 2 × 10^5^ cells per well and incubated with IFN-ɣ (100ng/ml). Untreated cells were used as a control. Following a 24 h incubation at 37 °C in a 5% CO_2_ atmosphere, expression levels of PD-L1 mRNA were evaluated by Real-Time (RT)-PCR. The levels of PD-L1, normalized to GAPDH and relative to HCC827^G/G^ cells, are plotted and expressed as mean ± SD of the results obtained in three independent experiments. (**P ≤ 0.01; ***P ≤ 0.001). (**B)** EGFR^mut^ HCC827^G/G^, H1975^G/G^, PC-9^G/G^ and EGFR^wt^ H1299^C/C^, H1703^C/C^ and H1437^C/C^ cells were seeded into 6-well plates at a density of 2 × 10^6^ cells per well and incubated with IFN-ɣ (100ng/ml). Untreated cells were used as a control. Following a 48 h incubation at 37 °C in a 5% CO_2_ atmosphere, cells were harvested. Cell lysates were analyzed by western blot with PD-L1-specific Ab. GAPDH was used as a loading control. Data are representative of the results obtained in three independent experiments (**left panel**). The levels of PD-L1, normalized to GAPDH and relative to HCC827^G/G^ cells, are plotted and expressed as mean ± SD of the results obtained in three independent experiments (**P ≤ 0.01; ***P ≤ 0.001) (**right panel**).

Additional file 6: **figure S5** Morphological changes and inhibition of proliferation by IFN-ɣ in NSCLC cell lines carrying different rs822336 genotype. EGFR^mut^ HCC827^G/G^, H1975^G/G^, PC-9^G/G^ and EGFR^wt^ H1299^C/C^, H1703^C/C^ and H1437^C/C^ cells were seeded into 6-well plates at a density of 2 × 10^6^ cells per well and incubated with IFN-ɣ (100ng/ml). Untreated cells were used as a control. Following a 48 h incubation at 37 °C in a 5% CO_2_ atmosphere, (**A**) three random regions from both untreated and treated cells were captured with inverted microscope (Bars: 50 mm); (**B**) cells were harvested and counted by 0.2% trypan blue exclusion test. Data are expressed as mean ± SD of the results obtained in three independent experiments.

Additional file 7: **figure S6** Characterization of HLA and β2m in NSCLC cell lines. EGFR^mut^ HCC827^G/G^, H1975^G/G^, PC-9^G/G^ and EGFR^wt^ H460^G/G^, A549^G/G^, H1299^C/C^, H1703^C/C^ and H1437^C/C^ cells were seeded into 6-well plates at the density of 2 × 10^6^ per well. Following a 24 h incubation at 37 °C in a 5% CO_2_ atmosphere, cells were harvested. (**A**) DNA was extracted and genotyped for HLA class I haplotypes utilizing PCR. (**B**) Cells were stained with HLA class I/II antigen- [mAb LGIII-147.4.1 (HLA-A), mAb B1.23.2 (HLA-B,C), mAb TP25.99.8.4 (HLA-A,B,C)], and β2m-specific (mAb LGII-612.14) mAbs. mAb MK2-23 was used as a specificity control. Cell staining was detected by Fluorescein isothiocyanate (FITC) anti-mouse IgG Ab by flow cytometry analysis. Data, expressed as mean fluorescence intensity (MFI), are representative of the results obtained in three independent experiments.

Additional file 8: **figure S7** Validation of TFs involved in the regulation of *PD-L1* gene expression based on rs822336 allele-specificity. H1975^G/G^ and H1299^C/C^ cells were seeded into T75 flasks at a density of 5 × 10^6^ cells. Following a 48 h incubation at 37 °C in a 5% CO_2_ atmosphere, DNA-pull down assay was performed with immobilized wt/mut oligos incubated with nuclear extracts on 4 distinct sample group combinations. Nuclear extract of H1299^C/C^ cells was incubated with the mut oligo; nuclear extract of H1299^C/C^ cells was incubated with the wt oligo; nuclear extract of H1975^G/G^ cells was incubated with the mut oligo; nuclear extract of H1975^G/G^ cells was incubated with wt oligo. Putative TFs of the rs822336 region of the *PD-L1* gene based on its allele-specificity were detected by LC-MS/MSVolcano plot shows a proteomic based comparison between different samples. Reported points indicate proteins that display both large magnitude fold-changes (x axis) and high statistical significance (y axis). Corresponding points to C/EBPβ and NFIC are highlighted.

Additional file 9: **supplementary file 2** List of the proteins identified by DNA pull-down assay and LC MS/MS performed incubating the H1975^G/G^ nuclear extract with wt oligo or H1299^C/C^ nuclear extract with mut oligo. The abundance ratio of the proteins in the two samples are also reported. *Abundance ratio of 100 indicates a protein detected only in H1299^C/C^+mut whereas a value of 0.01 indicates a protein detected only in H1975^G/G^+wt.

Additional file 10: **figure S8** Silencing of C/EBPβ and NFIC in EGFR^mut^ H1975^G/G^ and EGFR^wt^ H1299^C/C^ cell lines. Cells were seeded into 6-well plates at a density of 2 × 10^6^ cells per well. Following a 48 h of transfection at 37 °C in a 5% CO_2_ atmosphere with C/EBPβ - and NFIC-specific siRNAs cells were harvested and lysed. A siRNA-control was used as a control. Cell lysates were analyzed by western blot with C/EBPβ and NFIC-specific Abs. GAPDH was used as a loading control. Representative results are shown.

Additional file 11: **figure S9** EGFR^mut^ H1975^G/G^ and EGFR^wt^ H1299^C/C^ cells transduced with C/EBPβ- and NFIC-specific siRNAs or siRNA-controls were seeded into 24-well plates at a density of 2 × 10^5^ cells per well and incubated with IFN-ɣ (100ng/ml). Untreated cells were used as a control. Following a 24 h incubation at 37 °C in a 5% CO_2_ atmosphere, expression levels of NFIC, C/EBPβ and PD-L1 mRNA were evaluated by Real-Time (RT)-PCR. The levels of NFIC, C/EBPβ and PD-L1, normalized to GAPDH and relative to untreated siRNA-control of each analysed gene, are plotted and expressed as mean ± SD of the results obtained in three independent experiments (***P ≤ 0.001).

Additional file 12: **figure S10** Activation of IFN-ɣ pathway in NSCLC cell lines transfected with siRNAs. EGFR^mut^ H1975^G/G^ (left panel) and EGFR^wt^ H1299^C/C^ (right panel) cells were seeded into 6-well plates at a density of 2 × 10^6^ cells per well and incubated with IFN-ɣ (100ng/ml). Untreated cells were used as a control. Following a 48 h of incubation at 37 °C in a 5% CO_2_ atmosphere, cells transfected with the indicated siRNAs were harvested and lysed. Cell lysates were analyzed by western blot with p-STAT1 and STAT1-specific Abs. Representative results are shown. The levels of STAT1, normalized to GAPDH and relative to untreated siRNA-control as well as levels of p-STAT1 normalized to STAT1, are plotted below and expressed as mean ± SD of the results obtained in three independent experiments (***P ≤ 0.001).

## Data Availability

All data are available in the main text or the supplementary information. Any raw data supporting the conclusions of this article will be made available by the authors, without undue reservation.

## Abbreviations

ALK: Anaplastic lymphoma kinase
ATCC: American Type Culture Collection
APC: Allophycocyanin
β2m: β2-microglobulin
COPD: Chronic obstructive pulmonary disease
CR: Complete response
CTCAE: Common Terminology Criteria for Adverse Events
ECOG: Eastern Cooperative Oncology Group
EFI: European Federation of Immunogenetics
EGFR: Epidermal growth factor receptor
ESMO: European Society for Medical Oncology
FBS: Fetal bovin serum
FITC: Fluorescein isothiocyanate
GEP: Gene expression profile
HER2: human epidermal growth factor receptor 2
HLA: Human leukocyte antigen
HRP: Horseradish peroxidase
ICI: Immune checkpoint inhibitor
irAEs: Immune-related adverse events
IFN-γ: Interferon gamma
LDH: Lactate Dehydrogenase
mAb: Monoclonal antibody
MET: mesenchymal epithelial transition factor
NSCLC: Non-small cell lung cancer
NTRK1 and 2: Neurotrophic tyrosine receptor kinase 1 and 2
ORR: Objective response rate
OS: Overall survival
PBS: Phosphate-buffered saline
PCR: Polymerase chain reaction
PD: Progressive disease
PD-1: Programmed cell death-1
PD-L1: Programmed death-ligand 1
PFS: Progression-free survival
PR: Partial response
PS: Performance status
RECIST: Response Evaluation Criteria in Solid Tumours
RET: rearranged during transfection
SD: Stable disease
SSP: Sequence-specific primers
SNP: Single nucleotide polymorphism
T-Act: T Cell Trans Act
TF: Transcription factor
TFBS: Binding site for transcription factor
TCGA: The Cancer Genome Atlas
TMB: Tumor mutational burden
TME: Tumor microenvironment
TNFα: Tumor Necrosis Factor alpha
TPS: Tumor proportion score
