# Supplementary material for: rs822336 binding to C/EBPβ and NFIC modulates induction of PD-L1 expression and predicts anti-PD-1/PD-L1 therapy in advanced NSCLC
Source: Mol Cancer. 2024 Mar 25;23:63. doi: 10.1186/s12943-024-01976-2 (PMC10962156; doi:10.1186/s12943-024-01976-2)
Supplement: Supplementary file 1 — Additional file 1: supplementary file 1 Sequences of double-stranded oligos utilized (biotinylation at the 5′-end). [file 12943_2024_1976_MOESM1_ESM.docx]

**rs822336 binding to C/EBPβ and NFIC modulates induction of PD-L1 expression and predicts anti-PD-1/PD-L1 therapy in advanced NSCLC**

Giovanna Polcaro^1^†, Luigi Liguori^1,2^†, Valentina Manzo^3,4^, Annalisa Chianese^5^, Giuliana Donadio^6^, Alessandro Caputo^4,7^, Giosuè Scognamiglio^8^, Federica Dell’Annunziata^5^, Maddalena Langella^9^, Graziamaria Corbi^10^, Alessandro Ottaiano^11^, Marco Cascella^12^, Francesco Perri^13^, Margot De Marco^6^, Jessica Dal Col^6^, Giovanni Nassa^14^, Giorgio Giurato^14^, Pio Zeppa^4,7^, Amelia Filippelli^3,4^, Gianluigi Franci^4,15^, Fabrizio Dal Piaz^4,6^, Valeria Conti^3,4^*, Stefano Pepe^1,4^*, Francesco Sabbatino^1,4^*

† These authors contributed equally to this work

**Correspondence:**

Valeria Conti, Clinical Pharmacology Unit, Department of Medicine, Surgery and Dentistry, University of Salerno; Baronissi, 84081, Italy.

Email: vconti@unisa.it

Stefano Pepe, Oncology Unit, Department of Medicine, Surgery and Dentistry, University of Salerno; Baronissi, 84081, Italy.

Email: spepe@unisa.it

Francesco Sabbatino, Oncology Unit, Department of Medicine, Surgery and Dentistry, University of Salerno; Baronissi, 84081, Italy.

Email: fsabbatino@unisa.it

**Supplementary file 1** Sequences of double-stranded oligos utilized (biotinylation at the 5′-end).

| Mutation | Oligo sequences | bp | 5’ modification |
| --- | --- | --- | --- |
| G🡪 G (wt) | Forward: TTACTAATACGCAAATCACTGAGCAGCAAGCTGAGCAAATACCCTCAATTC  Reverse: AATGATTATGCGTTTAGTGACTCGTCGTTCGACTCGTTTATGGGAGTTAAG | 51 | biotin |
| G🡪C (mut) | Forward: TTACTAATACGCAAATCACTGAGCACCAAGCTGAGCAAATACCCTCAATTC  Reverse:  AATGATTATGCGTTTAGTGACTCGTGGTTCGACTCGTTTATGGGAGTTAAG | 51 | biotin |
| Scramble | Forward: ACCGGACTGATCGTACATAACTCGCAACCATACAATCCGATAATCAATATG  Reverse:  TGGCCTGACTAGCATGTATTGAGCGTTGGTATGTTAGGCTATTAGTTATAC | 51 | biotin |
