# Supplementary material for: rs822336 binding to C/EBPβ and NFIC modulates induction of PD-L1 expression and predicts anti-PD-1/PD-L1 therapy in advanced NSCLC
Source: Mol Cancer. 2024 Mar 25;23:63. doi: 10.1186/s12943-024-01976-2 (PMC10962156; doi:10.1186/s12943-024-01976-2)

**Figure S1** PFS and OS of advanced NSCLC patients treated with anti-PD-1/PD-L1 therapy. At a median follow-up of 41.46 months (range, 12.56-53.20 months) median PFS and OS were 3.52 months (**A**) and 8.53 months (**B**), respectively. PFS and OS analysis was performed using the Kaplan-Meier method.


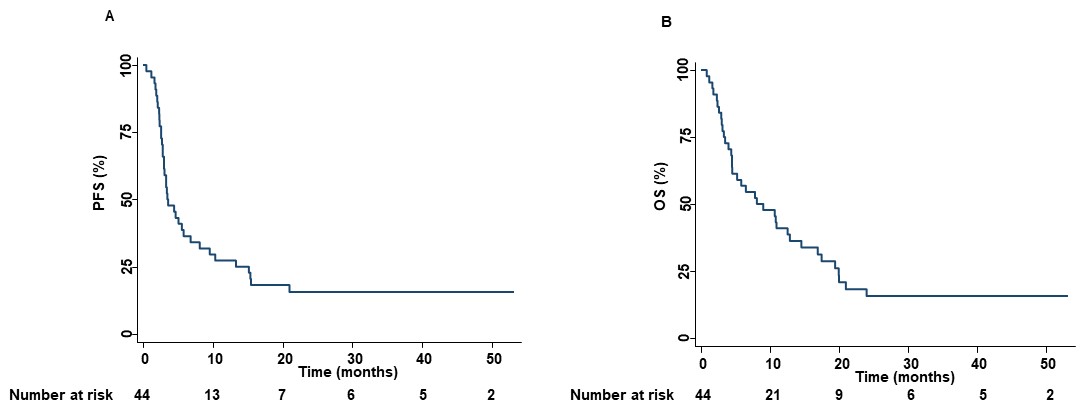

Supplement: Supplementary file 2 — Additional file 2: figure S1 PFS and OS of advanced NSCLC patients treated with anti-PD-1/PD-L1 therapy. At a median follow-up of 41.46 months (range, 12.56–53.20 months) median PFS and OS were 3.52 months (A) and 8.53 months (B), respectively. PFS and OS analysis was performed using the Kaplan-Meier method. [file 12943_2024_1976_MOESM2_ESM.docx]
