# Supplementary material for: rs822336 binding to C/EBPβ and NFIC modulates induction of PD-L1 expression and predicts anti-PD-1/PD-L1 therapy in advanced NSCLC
Source: Mol Cancer. 2024 Mar 25;23:63. doi: 10.1186/s12943-024-01976-2 (PMC10962156; doi:10.1186/s12943-024-01976-2)

**Figure S2** Association between rs2282055 or rs4143815 and clinical outcomes in advanced NSCLC patients treated with anti-PD-1/PD-L1 therapy. PFS (**A**) and OS (**B**) of NSCLC patients treated with anti-PD-1/PD-L1 therapy were stratified based on PD-L1 rs2282055 (**upper panel**) or rs4143815 (**bottom panel**) genotypes. PFS and OS were compared using the Kaplan-Meier method. Differences in patients’ survival were analyzed using a log-rang test. P < 0.05 was considered statistically significant


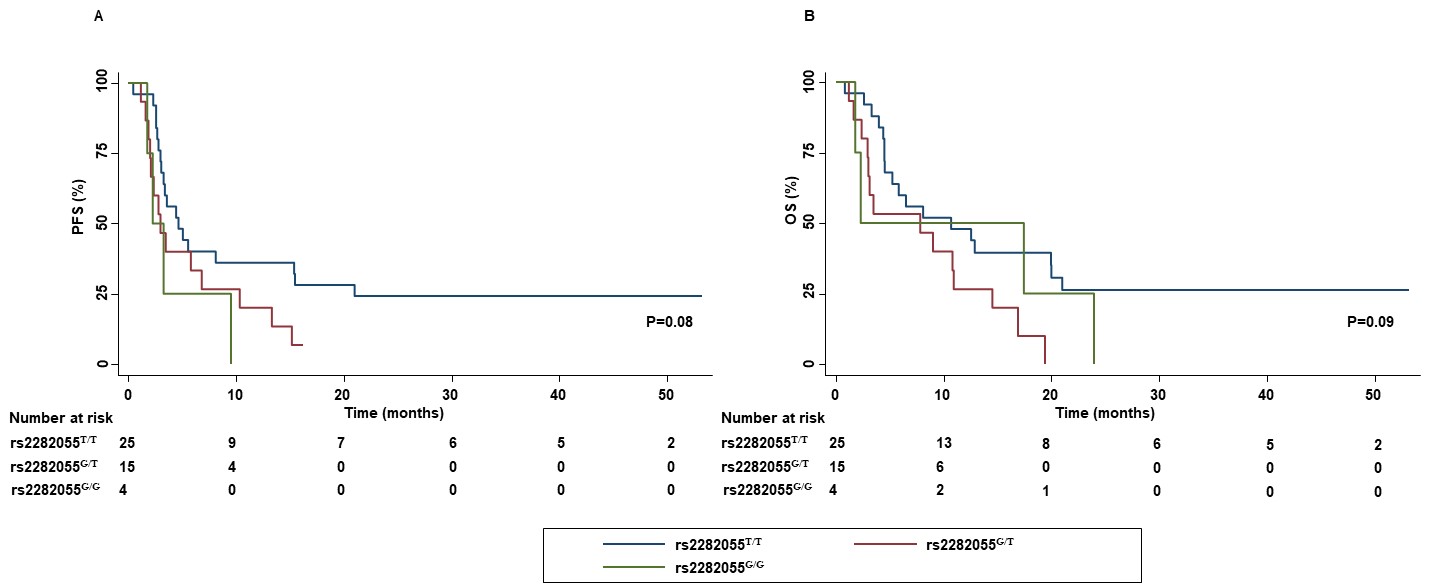


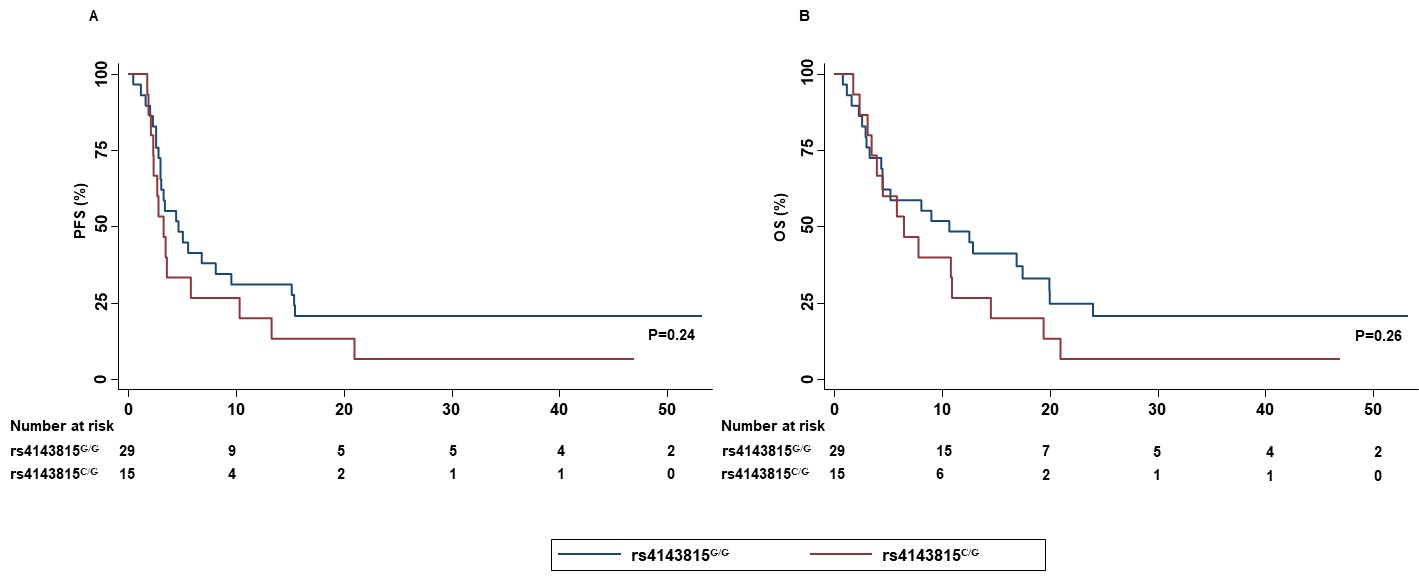

Supplement: Supplementary file 3 — Additional file 3: figure S2 Association between rs2282055 or rs4143815 and clinical outcomes in advanced NSCLC patients treated with anti-PD-1/PD-L1 therapy. PFS (A) and OS (B) of NSCLC patients treated with anti-PD-1/PD-L1 therapy were stratified based on PD-L1 rs2282055 (upper panel) or rs4143815 (bottom panel) genotypes. PFS and OS were compared using the Kaplan-Meier method. Differences in patients’ survival were analyzed using a log-rang test. P < 0.05 was considered statistically significant. [file 12943_2024_1976_MOESM3_ESM.docx]
