# Supplementary material for: rs822336 binding to C/EBPβ and NFIC modulates induction of PD-L1 expression and predicts anti-PD-1/PD-L1 therapy in advanced NSCLC
Source: Mol Cancer. 2024 Mar 25;23:63. doi: 10.1186/s12943-024-01976-2 (PMC10962156; doi:10.1186/s12943-024-01976-2)

**Figure S3** Characterization of human EGFR^mut^ and EGFR^wt^ NSCLC cell lines. HCC827, H1975, PC-9, H1299, H1703 and H1437 cells were seeded into 6-well plates at the density of 2×10^6^ per well. Following a 24h incubation at 37°C in a 5% CO_2_ atmosphere, cells were harvested. DNA was extracted and genotyped for rs2282055 or rs4143815 *PD-L1* SNPs utilizing PCR.


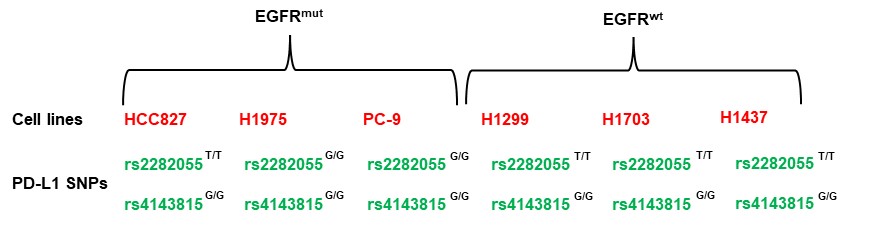

Supplement: Supplementary file 4 — Additional file 4: figure S3 Characterization of human EGFRmut and EGFRwt NSCLC cell lines. HCC827, H1975, PC-9, H1299, H1703 and H1437 cells were seeded into 6-well plates at the density of 2 × 10^6^ per well. Following a 24 h incubation at 37 °C in a 5% CO2 atmosphere, cells were harvested. DNA was extracted and genotyped for rs2282055 or rs4143815 PD-L1 SNPs utilizing PCR. [file 12943_2024_1976_MOESM4_ESM.docx]
