# Supplementary material for: rs822336 binding to C/EBPβ and NFIC modulates induction of PD-L1 expression and predicts anti-PD-1/PD-L1 therapy in advanced NSCLC
Source: Mol Cancer. 2024 Mar 25;23:63. doi: 10.1186/s12943-024-01976-2 (PMC10962156; doi:10.1186/s12943-024-01976-2)

**Figure S4** Modulation by IFN-ɣ of PD-L1 expression in NSCLC cell lines carrying different rs822336 genotype. (**A**) EGFR^mut^ HCC827^G/G^, H1975^G/G^, PC-9^G/G^ and EGFR^wt^ H1299^C/C^, H1703^C/C^ and H1437^C/C^ cells were seeded into 24-well plates at a density of 2×10^5^ cells per well and incubated with IFN-ɣ (100ng/ml). Untreated cells were used as a control. Following a 24h incubation at 37°C in a 5% CO_2_ atmosphere, expression levels of PD-L1 mRNA were evaluated by Real-Time (RT)-PCR. The levels of PD-L1, normalized to GAPDH and relative to HCC827^G/G^ cells, are plotted and expressed as mean ± SD of the results obtained in three independent experiments. (**P ≤ 0.01; ***P ≤ 0.001). (**B)** EGFR^mut^ HCC827^G/G^, H1975^G/G^, PC-9^G/G^ and EGFR^wt^ H1299^C/C^, H1703^C/C^ and H1437^C/C^ cells were seeded into 6-well plates at a density of 2×10^6^ cells per well and incubated with IFN-ɣ (100ng/ml). Untreated cells were used as a control. Following a 48h incubation at 37°C in a 5% CO_2_ atmosphere, cells were harvested. Cell lysates were analyzed by western blot with PD-L1-specific Ab. GAPDH was used as a loading control. Data are representative of the results obtained in three independent experiments (**left panel**). The levels of PD-L1, normalized to GAPDH and relative to HCC827^G/G^ cells, are plotted and expressed as mean ± SD of the results obtained in three independent experiments (**P ≤ 0.01; ***P ≤ 0.001) (**right panel**).


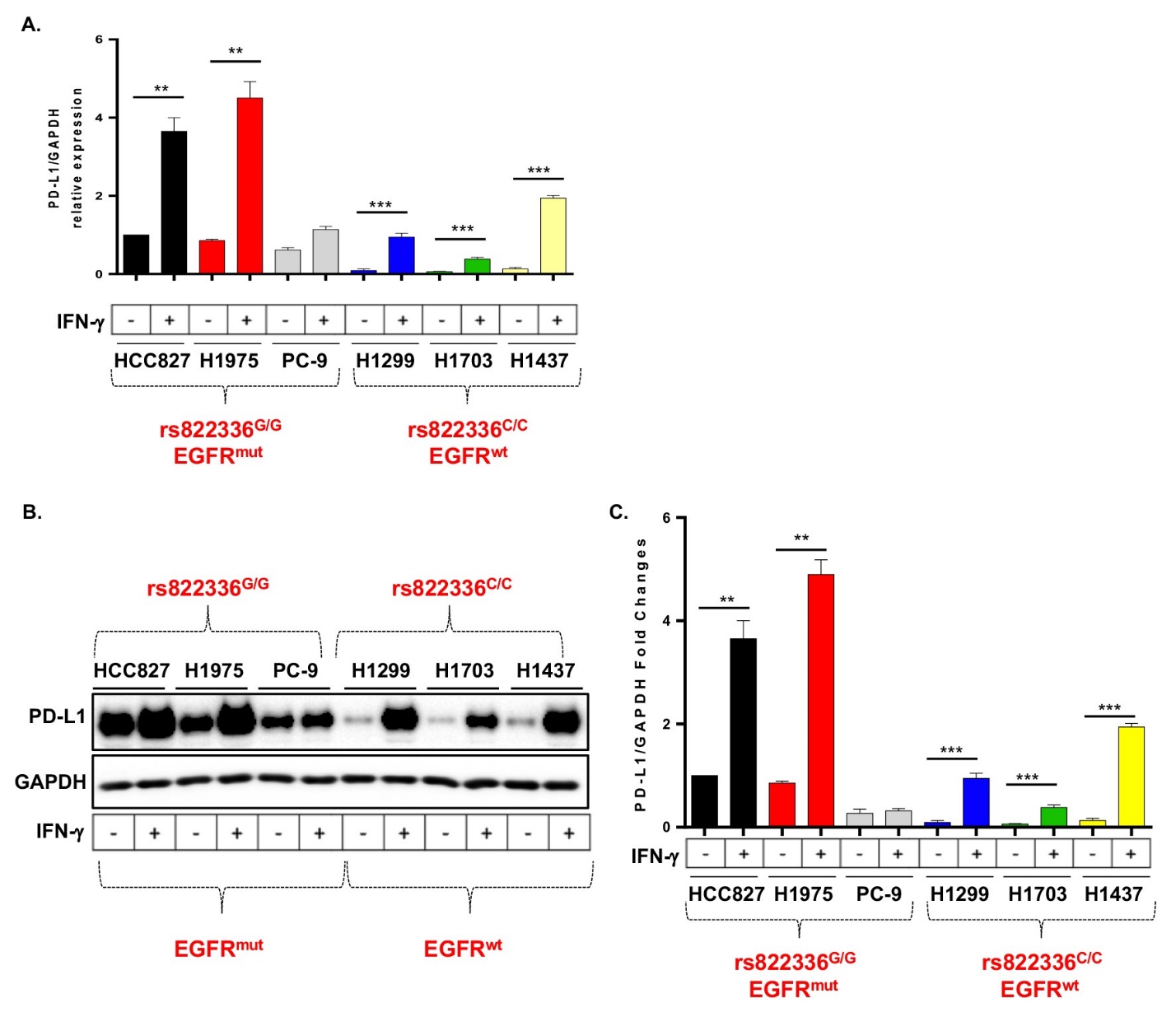

Supplement: Supplementary file 5 — Additional file 5: figure S4 Modulation by IFN-ɣ of PD-L1 expression in NSCLC cell lines carrying different rs822336 genotype. (A) EGFRmut HCC827G/G, H1975G/G, PC-9G/G and EGFRwt H1299C/C, H1703C/C and H1437C/C cells were seeded into 24-well plates at a density of 2 × 10^5^ cells per well and incubated with IFN-ɣ (100ng/ml). Untreated cells were used as a control. Following a 24 h incubation at 37 °C in a 5% CO2 atmosphere, expression levels of PD-L1 mRNA were evaluated by Real-Time (RT)-PCR. The levels of PD-L1, normalized to GAPDH and relative to HCC827G/G cells, are plotted and expressed as mean ± SD of the results obtained in three independent experiments. (**P ≤ 0.01; ***P ≤ 0.001). (B) EGFRmut HCC827G/G, H1975G/G, PC-9G/G and EGFRwt H1299C/C, H1703C/C and H1437C/C cells were seeded into 6-well plates at a density of 2 × 10^6^ cells per well and incubated with IFN-ɣ (100ng/ml). Untreated cells were used as a control. Following a 48 h incubation at 37 °C in a 5% CO2 atmosphere, cells were harvested. Cell lysates were analyzed by western blot with PD-L1-specific Ab. GAPDH was used as a loading control. Data are representative of the results obtained in three independent experiments (left panel). The levels of PD-L1, normalized to GAPDH and relative to HCC827G/G cells, are plotted and expressed as mean ± SD of the results obtained in three independent experiments (**P ≤ 0.01; ***P ≤ 0.001) (right panel). [file 12943_2024_1976_MOESM5_ESM.docx]
