# Supplementary material for: rs822336 binding to C/EBPβ and NFIC modulates induction of PD-L1 expression and predicts anti-PD-1/PD-L1 therapy in advanced NSCLC
Source: Mol Cancer. 2024 Mar 25;23:63. doi: 10.1186/s12943-024-01976-2 (PMC10962156; doi:10.1186/s12943-024-01976-2)

**Figure S5** Morphological changes and inhibition of proliferation by IFN-ɣ in NSCLC cell lines carrying different rs822336 genotype. EGFR^mut^ HCC827^G/G^, H1975^G/G^, PC-9^G/G^ and EGFR^wt^ H1299^C/C^, H1703^C/C^ and H1437^C/C^ cells were seeded into 6-well plates at a density of 2×10^6^ cells per well and incubated with IFN-ɣ (100ng/ml). Untreated cells were used as a control. Following a 48h incubation at 37°C in a 5% CO_2_ atmosphere, (**A**) three random regions from both untreated and treated cells were captured with inverted microscope (Bars: 50 mm); (**B**) cells were harvested and counted by 0.2% trypan blue exclusion test. Data are expressed as mean ± SD of the results obtained in three independent experiments.


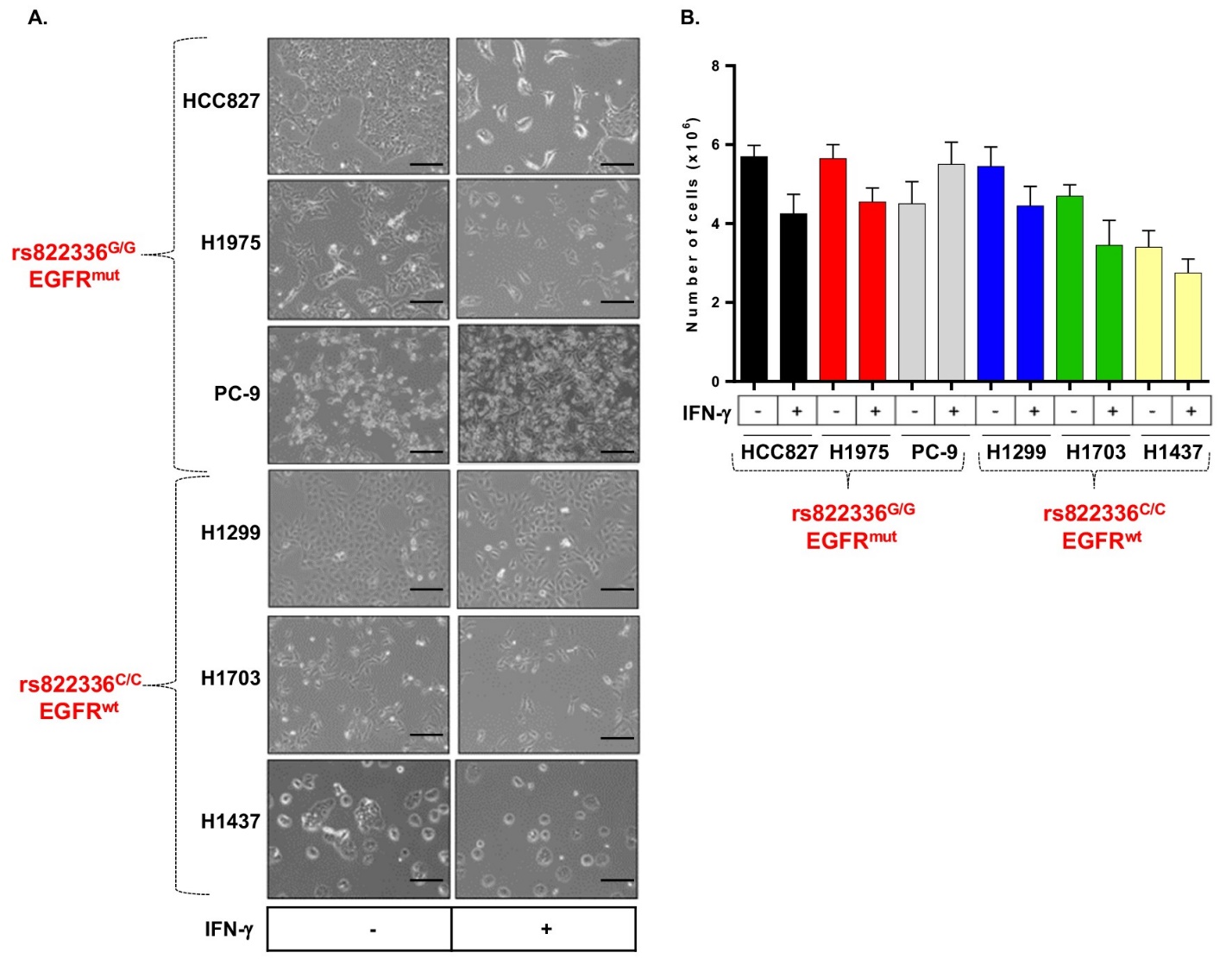

Supplement: Supplementary file 6 — Additional file 6: figure S5 Morphological changes and inhibition of proliferation by IFN-ɣ in NSCLC cell lines carrying different rs822336 genotype. EGFRmut HCC827G/G, H1975G/G, PC-9G/G and EGFRwt H1299C/C, H1703C/C and H1437C/C cells were seeded into 6-well plates at a density of 2 × 10^6^ cells per well and incubated with IFN-ɣ (100ng/ml). Untreated cells were used as a control. Following a 48 h incubation at 37 °C in a 5% CO2 atmosphere, (A) three random regions from both untreated and treated cells were captured with inverted microscope (Bars: 50 mm); (B) cells were harvested and counted by 0.2% trypan blue exclusion test. Data are expressed as mean ± SD of the results obtained in three independent experiments. [file 12943_2024_1976_MOESM6_ESM.docx]
