# Supplementary material for: rs822336 binding to C/EBPβ and NFIC modulates induction of PD-L1 expression and predicts anti-PD-1/PD-L1 therapy in advanced NSCLC
Source: Mol Cancer. 2024 Mar 25;23:63. doi: 10.1186/s12943-024-01976-2 (PMC10962156; doi:10.1186/s12943-024-01976-2)

**Figure S6** Characterization of HLA and β2m in NSCLC cell lines. EGFR^mut^ HCC827^G/G^, H1975^G/G^, PC-9^G/G^ and EGFR^wt^ H460^G/G^, A549^G/G^, H1299^C/C^, H1703^C/C^ and H1437^C/C^ cells were seeded into 6-well plates at the density of 2×10^6^ per well. Following a 24h incubation at 37°C in a 5% CO_2_ atmosphere, cells were harvested. (**A**) DNA was extracted and genotyped for HLA class I haplotypes utilizing PCR. (**B**) Cells were stained with HLA class I/II antigen- [mAb LGIII-147.4.1 (HLA-A), mAb B1.23.2 (HLA-B,C), mAb TP25.99.8.4 (HLA-A,B,C)], and β2m-specific (mAb LGII-612.14) mAbs. mAb MK2-23 was used as a specificity control. Cell staining was detected by Fluorescein isothiocyanate (FITC) anti-mouse IgG Ab by flow cytometry analysis. Data, expressed as mean fluorescence intensity (MFI), are representative of the results obtained in three independent experiments.


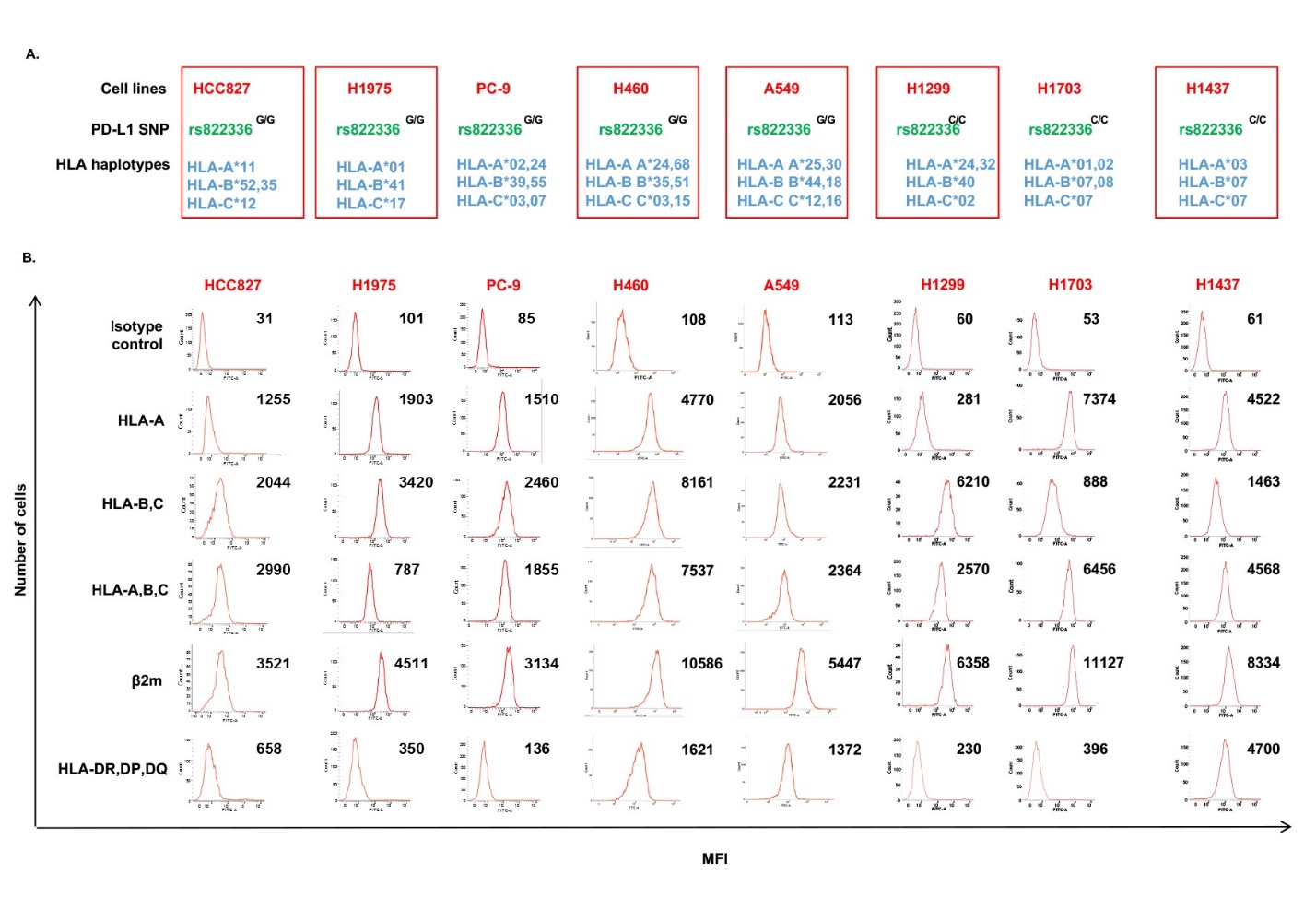

Supplement: Supplementary file 7 — Additional file 7: figure S6 Characterization of HLA and β2m in NSCLC cell lines. EGFRmut HCC827G/G, H1975G/G, PC-9G/G and EGFRwt H460G/G, A549G/G, H1299C/C, H1703C/C and H1437C/C cells were seeded into 6-well plates at the density of 2 × 10^6^ per well. Following a 24 h incubation at 37 °C in a 5% CO2 atmosphere, cells were harvested. (A) DNA was extracted and genotyped for HLA class I haplotypes utilizing PCR. (B) Cells were stained with HLA class I/II antigen- [mAb LGIII-147.4.1 (HLA-A), mAb B1.23.2 (HLA-B,C), mAb TP25.99.8.4 (HLA-A,B,C)], and β2m-specific (mAb LGII-612.14) mAbs. mAb MK2-23 was used as a specificity control. Cell staining was detected by Fluorescein isothiocyanate (FITC) anti-mouse IgG Ab by flow cytometry analysis. Data, expressed as mean fluorescence intensity (MFI), are representative of the results obtained in three independent experiments. [file 12943_2024_1976_MOESM7_ESM.docx]
