# Supplementary material for: rs822336 binding to C/EBPβ and NFIC modulates induction of PD-L1 expression and predicts anti-PD-1/PD-L1 therapy in advanced NSCLC
Source: Mol Cancer. 2024 Mar 25;23:63. doi: 10.1186/s12943-024-01976-2 (PMC10962156; doi:10.1186/s12943-024-01976-2)

**Figure S7** Validation of TFs involved in the regulation of *PD-L1* gene expression based on rs822336 allele-specificity. H1975^G/G^ and H1299^C/C^ cells were seeded into T75 flasks at a density of 5×10^6^ cells. Following a 48h incubation at 37°C in a 5% CO_2_ atmosphere, DNA-pull down assay was performed with immobilized wt/mut oligos incubated with nuclear extracts on 4 distinct sample group combinations. Nuclear extract of H1299^C/C^ cells was incubated with the mut oligo; nuclear extract of H1299^C/C^ cells was incubated with the wt oligo; nuclear extract of H1975^G/G^ cells was incubated with the mut oligo; nuclear extract of H1975^G/G^ cells was incubated with wt oligo. Putative TFs of the rs822336 region of the *PD-L1* gene based on its allele-specificity were detected by LC-MS/MSVolcano plot shows a proteomic based comparison between different samples. Reported points indicate proteins that display both large magnitude fold-changes (x axis) and high statistical significance (y axis). Corresponding points to C/EBPβ and NFIC are highlighted.


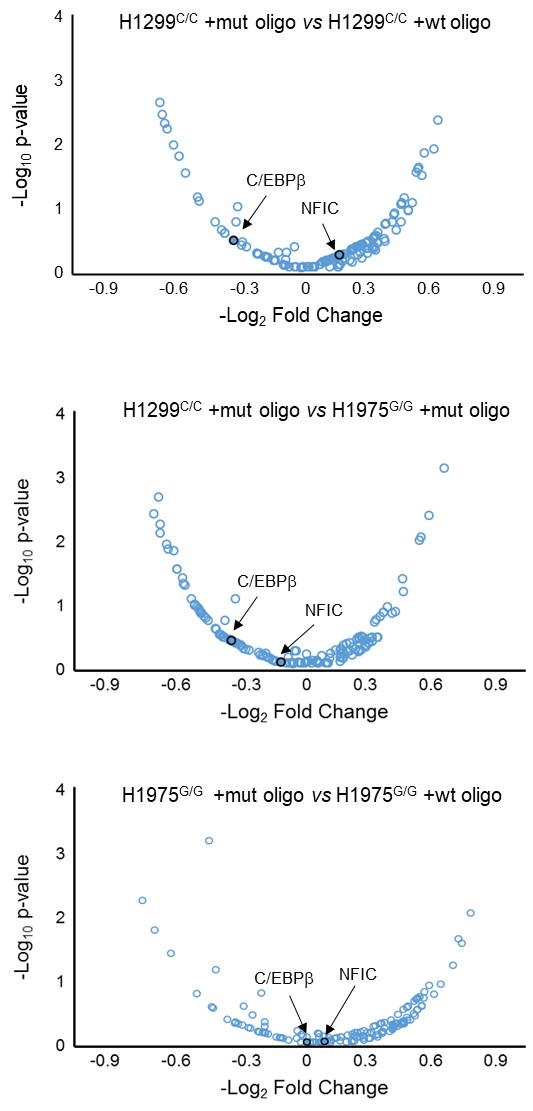

Supplement: Supplementary file 8 — Additional file 8: figure S7 Validation of TFs involved in the regulation of PD-L1 gene expression based on rs822336 allele-specificity. H1975G/G and H1299C/C cells were seeded into T75 flasks at a density of 5 × 10^6^ cells. Following a 48 h incubation at 37 °C in a 5% CO2 atmosphere, DNA-pull down assay was performed with immobilized wt/mut oligos incubated with nuclear extracts on 4 distinct sample group combinations. Nuclear extract of H1299C/C cells was incubated with the mut oligo; nuclear extract of H1299C/C cells was incubated with the wt oligo; nuclear extract of H1975G/G cells was incubated with the mut oligo; nuclear extract of H1975G/G cells was incubated with wt oligo. Putative TFs of the rs822336 region of the PD-L1 gene based on its allele-specificity were detected by LC-MS/MSVolcano plot shows a proteomic based comparison between different samples. Reported points indicate proteins that display both large magnitude fold-changes (x axis) and high statistical significance (y axis). Corresponding points to C/EBPβ and NFIC are highlighted. [file 12943_2024_1976_MOESM8_ESM.docx]
