# Supplementary material for: rs822336 binding to C/EBPβ and NFIC modulates induction of PD-L1 expression and predicts anti-PD-1/PD-L1 therapy in advanced NSCLC
Source: Mol Cancer. 2024 Mar 25;23:63. doi: 10.1186/s12943-024-01976-2 (PMC10962156; doi:10.1186/s12943-024-01976-2)
Supplement: Supplementary file 9 — Additional file 9: supplementary file 2 List of the proteins identified by DNA pull-down assay and LC MS/MS performed incubating the H1975G/G nuclear extract with wt oligo or H1299C/C nuclear extract with mut oligo. The abundance ratio of the proteins in the two samples are also reported. *Abundance ratio of 100 indicates a protein detected only in H1299C/C+mut whereas a value of 0.01 indicates a protein detected only in H1975G/G+wt. [file 12943_2024_1976_MOESM9_ESM.docx]

**Supplementary file 2** List of the proteins identified by DNA pull-down assay and LC MS/MS performed incubating the H1975^G/G^ nuclear extract with wt oligo or H1299^C/C^ nuclear extract with mut oligo. The abundance ratio of the proteins in the two samples are also reported. *Abundance ratio of 100 indicates a protein detected only in H1299^C/C^+mut whereas a value of 0.01 indicates a protein detected only in H1975^G/G^+wt.

| Accession | Description | Abundance Ratio:  (H1299^C/C^+mut) / (H1975^G/G^+wt)* |
| --- | --- | --- |
| A0A494C0G1 | Phosphoacetylglucosamine mutase OS=Homo sapiens OX=9606 GN=PGM3 PE=1 SV=1 | 100 |
| Q13084 | 39S ribosomal protein L28, mitochondrial OS=Homo sapiens OX=9606 GN=MRPL28 PE=1 SV=4 | 100 |
| Q9UMY1 | Nucleolar protein 7 OS=Homo sapiens OX=9606 GN=NOL7 PE=1 SV=2 | 100 |
| Q9Y2T7 | Y-box-binding protein 2 OS=Homo sapiens OX=9606 GN=YBX2 PE=1 SV=2 | 100 |
| K7EJL1 | AP-1 complex subunit mu-1 OS=Homo sapiens OX=9606 GN=AP1M1 PE=1 SV=1 | 100 |
| Q9Y6M9 | NADH dehydrogenase [ubiquinone] 1 beta subcomplex subunit 9 OS=Homo sapiens OX=9606 GN=NDUFB9 PE=1 SV=3 | 100 |
| E9PFN5 | Glutathione S-transferase kappa OS=Homo sapiens OX=9606 GN=GSTK1 PE=1 SV=1 | 100 |
| A0A0A0MSQ0 | Plastin-3 OS=Homo sapiens OX=9606 GN=PLS3 PE=1 SV=1 | 100 |
| A0A3B3ISK9 | Malonate--CoA ligase ACSF3, mitochondrial OS=Homo sapiens OX=9606 GN=ACSF3 PE=1 SV=1 | 100 |
| E7EMC7 | Sequestosome-1 OS=Homo sapiens OX=9606 GN=SQSTM1 PE=1 SV=1 | 100 |
| F6RFD5 | Actin-depolymerizing factor OS=Homo sapiens OX=9606 GN=DSTN PE=1 SV=1 | 100 |
| F8WDN7 | Phosphatidylinositol-3-phosphatase SAC1 OS=Homo sapiens OX=9606 GN=SACM1L PE=1 SV=1 | 100 |
| Q9NWK9 | Box C/D snoRNA protein 1 OS=Homo sapiens OX=9606 GN=ZNHIT6 PE=1 SV=1 | 100 |
| Q96AE4 | Far upstream element-binding protein 1 OS=Homo sapiens OX=9606 GN=FUBP1 PE=1 SV=3 | 12.3 |
| O43324 | Eukaryotic translation elongation factor 1 epsilon-1 OS=Homo sapiens OX=9606 GN=EEF1E1 PE=1 SV=1 | 11.95 |
| F8WEJ5 | Asparagine synthetase [glutamine-hydrolyzing] OS=Homo sapiens OX=9606 GN=ASNS PE=1 SV=1 | 10.88 |
| F5H6I7 | Atlastin-3 OS=Homo sapiens OX=9606 GN=ATL3 PE=1 SV=1 | 10.83 |
| Q9H7B2 | Ribosome production factor 2 homolog OS=Homo sapiens OX=9606 GN=RPF2 PE=1 SV=2 | 10.44 |
| P47929 | Galectin-7 OS=Homo sapiens OX=9606 GN=LGALS7 PE=1 SV=2 | 9.75 |
| Q86XP3 | ATP-dependent RNA helicase DDX42 OS=Homo sapiens OX=9606 GN=DDX42 PE=1 SV=1 | 9.27 |
| P05109 | Protein S100-A8 OS=Homo sapiens OX=9606 GN=S100A8 PE=1 SV=1 | 8.99 |
| Q96LJ7 | Dehydrogenase/reductase SDR family member 1 OS=Homo sapiens OX=9606 GN=DHRS1 PE=1 SV=1 | 8.78 |
| Q16891 | MICOS complex subunit MIC60 OS=Homo sapiens OX=9606 GN=IMMT PE=1 SV=1 | 8.41 |
| F8WDR2 | tRNA (adenine(58)-N(1))-methyltransferase OS=Homo sapiens OX=9606 GN=TRMT61B PE=1 SV=1 | 8.33 |
| A0A0A0MTQ5 | ATP synthase subunit ATP5MPL, mitochondrial OS=Homo sapiens OX=9606 GN=ATP5MPL PE=1 SV=1 | 8.03 |
| A6ND22 | 28S ribosomal protein S16, mitochondrial OS=Homo sapiens OX=9606 GN=MRPS16 PE=1 SV=1 | 7.96 |
| P84243 | Histone H3.3 OS=Homo sapiens OX=9606 GN=H3-3A PE=1 SV=2 | 7.69 |
| P13804 | Electron transfer flavoprotein subunit alpha, mitochondrial OS=Homo sapiens OX=9606 GN=ETFA PE=1 SV=1 | 7.32 |
| G3V0E4 | Beta-MPP OS=Homo sapiens OX=9606 GN=PMPCB PE=1 SV=1 | 7.23 |
| P51970 | NADH dehydrogenase [ubiquinone] 1 alpha subcomplex subunit 8 OS=Homo sapiens OX=9606 GN=NDUFA8 PE=1 SV=3 | 7.14 |
| Q6P1L8 | 39S ribosomal protein L14, mitochondrial OS=Homo sapiens OX=9606 GN=MRPL14 PE=1 SV=1 | 6.75 |
| E9PEJ4 | Acetyltransferase component of pyruvate dehydrogenase complex OS=Homo sapiens OX=9606 GN=DLAT PE=1 SV=1 | 6.71 |
| P11766 | Alcohol dehydrogenase class-3 OS=Homo sapiens OX=9606 GN=ADH5 PE=1 SV=4 | 6.46 |
| Q9HC36 | rRNA methyltransferase 3, mitochondrial OS=Homo sapiens OX=9606 GN=MRM3 PE=1 SV=2 | 6.27 |
| P23368 | NAD-dependent malic enzyme, mitochondrial OS=Homo sapiens OX=9606 GN=ME2 PE=1 SV=1 | 6.26 |
| A0A087WUX8 | MICOS complex subunit OS=Homo sapiens OX=9606 GN=APOOL PE=1 SV=1 | 6.15 |
| P30419 | Glycylpeptide N-tetradecanoyltransferase 1 OS=Homo sapiens OX=9606 GN=NMT1 PE=1 SV=2 | 6.09 |
| A0A0D9SF54 | Spectrin alpha chain, non-erythrocytic 1 OS=Homo sapiens OX=9606 GN=SPTAN1 PE=1 SV=1 | 6.07 |
| Q6P087 | Mitochondrial mRNA pseudouridine synthase RPUSD3 OS=Homo sapiens OX=9606 GN=RPUSD3 PE=1 SV=3 | 6.01 |
| Q9BQ52 | Zinc phosphodiesterase ELAC protein 2 OS=Homo sapiens OX=9606 GN=ELAC2 PE=1 SV=2 | 5.92 |
| Q86TX2 | Acyl-coenzyme A thioesterase 1 OS=Homo sapiens OX=9606 GN=ACOT1 PE=1 SV=1 | 5.38 |
| P32322 | Pyrroline-5-carboxylate reductase 1, mitochondrial OS=Homo sapiens OX=9606 GN=PYCR1 PE=1 SV=2 | 4.68 |
| P82912 | 28S ribosomal protein S11, mitochondrial OS=Homo sapiens OX=9606 GN=MRPS11 PE=1 SV=2 | 4.03 |
| Q9HB71 | Calcyclin-binding protein OS=Homo sapiens OX=9606 GN=CACYBP PE=1 SV=2 | 2.4 |
| A0A0S2Z6F0 | Histocompatibility 13 isoform 2 OS=Homo sapiens OX=9606 GN=HM13 PE=1 SV=1 | 2.38 |
| P47985 | Cytochrome b-c1 complex subunit Rieske, mitochondrial OS=Homo sapiens OX=9606 GN=UQCRFS1 PE=1 SV=2 | 2.37 |
| Q8IV48 | 3'-5' exoribonuclease 1 OS=Homo sapiens OX=9606 GN=ERI1 PE=1 SV=3 | 2.32 |
| O00422 | Histone deacetylase complex subunit SAP18 OS=Homo sapiens OX=9606 GN=SAP18 PE=1 SV=1 | 2.22 |
| K7EP06 | RG7MT1 OS=Homo sapiens OX=9606 GN=RNMT PE=1 SV=1 | 2.2 |
| O15258 | Protein RER1 OS=Homo sapiens OX=9606 GN=RER1 PE=1 SV=1 | 2.09 |
| P34932 | Heat shock 70 kDa protein 4 OS=Homo sapiens OX=9606 GN=HSPA4 PE=1 SV=4 | 2.08 |
| Q9Y676 | 28S ribosomal protein S18b, mitochondrial OS=Homo sapiens OX=9606 GN=MRPS18B PE=1 SV=1 | 2.05 |
| Q86U42 | Polyadenylate-binding protein 2 OS=Homo sapiens OX=9606 GN=PABPN1 PE=1 SV=3 | 2.03 |
| O00303 | Eukaryotic translation initiation factor 3 subunit F OS=Homo sapiens OX=9606 GN=EIF3F PE=1 SV=1 | 2.01 |
| Q96I63 | ATP-dependent zinc metalloprotease YME1L1 OS=Homo sapiens OX=9606 GN=YME1L1 PE=1 SV=1 | 2.01 |
| P62308 | Small nuclear ribonucleoprotein G OS=Homo sapiens OX=9606 GN=SNRPG PE=1 SV=1 | 1.97 |
| Q96DP5 | Methionyl-tRNA formyltransferase, mitochondrial OS=Homo sapiens OX=9606 GN=MTFMT PE=1 SV=2 | 1.91 |
| Q8WU90 | Zinc finger CCCH domain-containing protein 15 OS=Homo sapiens OX=9606 GN=ZC3H15 PE=1 SV=1 | 1.9 |
| O00267 | Transcription elongation factor SPT5 OS=Homo sapiens OX=9606 GN=SUPT5H PE=1 SV=1 | 1.89 |
| B4DXZ6 | Fragile X mental retardation syndrome-related protein 1 OS=Homo sapiens OX=9606 GN=FXR1 PE=1 SV=1 | 1.89 |
| Q9Y3Z3 | Deoxynucleoside triphosphate triphosphohydrolase SAMHD1 OS=Homo sapiens OX=9606 GN=SAMHD1 PE=1 SV=2 | 1.89 |
| P78357 | Contactin-associated protein 1 OS=Homo sapiens OX=9606 GN=CNTNAP1 PE=1 SV=1 | 1.72 |
| Q5SRN1 | Pre-mRNA-processing factor 17 OS=Homo sapiens OX=9606 GN=CDC40 PE=1 SV=1 | 1.68 |
| A0A3B3IU46 | RNA guanine-7 methyltransferase-activating subunit-like (pseudogene) OS=Homo sapiens OX=9606 GN=RAMACL PE=4 SV=1 | 1.64 |
| Q7Z7F7 | 39S ribosomal protein L55, mitochondrial OS=Homo sapiens OX=9606 GN=MRPL55 PE=1 SV=1 | 1.62 |
| Q9Y2Q9 | 28S ribosomal protein S28, mitochondrial OS=Homo sapiens OX=9606 GN=MRPS28 PE=1 SV=1 | 1.57 |
| P62888 | 60S ribosomal protein L30 OS=Homo sapiens OX=9606 GN=RPL30 PE=1 SV=2 | 1.56 |
| H0YHX9 | Nascent polypeptide-associated complex subunit alpha OS=Homo sapiens OX=9606 GN=NACA PE=1 SV=2 | 1.49 |
| Q7Z739 | YTH domain-containing family protein 3 OS=Homo sapiens OX=9606 GN=YTHDF3 PE=1 SV=1 | 1.48 |
| A0A087WUE4 | Leukocyte receptor cluster member 8 OS=Homo sapiens OX=9606 GN=LENG8 PE=1 SV=1 | 1.47 |
| Q8IX01 | SURP and G-patch domain-containing protein 2 OS=Homo sapiens OX=9606 GN=SUGP2 PE=1 SV=2 | 1.45 |
| P55795 | Heterogeneous nuclear ribonucleoprotein H2 OS=Homo sapiens OX=9606 GN=HNRNPH2 PE=1 SV=1 | 1.41 |
| Q5T7U1 | General transcription factor 3C polypeptide 5 OS=Homo sapiens OX=9606 GN=GTF3C5 PE=1 SV=1 | 1.39 |
| P78417 | Glutathione S-transferase omega-1 OS=Homo sapiens OX=9606 GN=GSTO1 PE=1 SV=2 | 1.34 |
| Q9H0L4 | Cleavage stimulation factor subunit 2 tau variant OS=Homo sapiens OX=9606 GN=CSTF2T PE=1 SV=1 | 1.32 |
| P33991 | DNA replication licensing factor MCM4 OS=Homo sapiens OX=9606 GN=MCM4 PE=1 SV=5 | 1.31 |
| Q13247 | Serine/arginine-rich splicing factor 6 OS=Homo sapiens OX=9606 GN=SRSF6 PE=1 SV=2 | 1.29 |
| P62937 | Peptidyl-prolyl cis-trans isomerase A OS=Homo sapiens OX=9606 GN=PPIA PE=1 SV=2 | 1.25 |
| A0A0U1RRK1 | Calcium uptake protein 1, mitochondrial OS=Homo sapiens OX=9606 GN=MICU1 PE=1 SV=1 | 1.25 |
| P51659 | Peroxisomal multifunctional enzyme type 2 OS=Homo sapiens OX=9606 GN=HSD17B4 PE=1 SV=3 | 1.21 |
| P16989 | Y-box-binding protein 3 OS=Homo sapiens OX=9606 GN=YBX3 PE=1 SV=4 | 1.17 |
| P61254 | 60S ribosomal protein L26 OS=Homo sapiens OX=9606 GN=RPL26 PE=1 SV=1 | 1.12 |
| O75915 | PRA1 family protein 3 OS=Homo sapiens OX=9606 GN=ARL6IP5 PE=1 SV=1 | 1.11 |
| Q14258 | E3 ubiquitin/ISG15 ligase TRIM25 OS=Homo sapiens OX=9606 GN=TRIM25 PE=1 SV=2 | 1.09 |
| P08651 | Nuclear factor 1 C-type OS=Homo sapiens OX=9606 GN=NFIC | 1.09 |
| P61106 | Ras-related protein Rab-14 OS=Homo sapiens OX=9606 GN=RAB14 PE=1 SV=4 | 1.05 |
| A0A087WYW6 | Coiled-coil domain-containing protein 47 OS=Homo sapiens OX=9606 GN=CCDC47 PE=1 SV=1 | 1.05 |
| Q9Y295 | Developmentally-regulated GTP-binding protein 1 OS=Homo sapiens OX=9606 GN=DRG1 PE=1 SV=1 | 1.05 |
| P17858 | ATP-dependent 6-phosphofructokinase, liver type OS=Homo sapiens OX=9606 GN=PFKL PE=1 SV=6 | 1.01 |
| Q86SQ0 | Pleckstrin homology-like domain family B member 2 OS=Homo sapiens OX=9606 GN=PHLDB2 PE=1 SV=2 | 0.96 |
| Q9UJA5 | tRNA (adenine(58)-N(1))-methyltransferase non-catalytic subunit TRM6 OS=Homo sapiens OX=9606 GN=TRMT6 PE=1 SV=1 | 0.95 |
| P32780 | General transcription factor IIH subunit 1 OS=Homo sapiens OX=9606 GN=GTF2H1 PE=1 SV=1 | 0.94 |
| O43823 | A-kinase anchor protein 8 OS=Homo sapiens OX=9606 GN=AKAP8 PE=1 SV=1 | 0.88 |
| Q9UKA9 | Polypyrimidine tract-binding protein 2 OS=Homo sapiens OX=9606 GN=PTBP2 PE=1 SV=1 | 0.88 |
| Q9NUW8 | Tyrosyl-DNA phosphodiesterase 1 OS=Homo sapiens OX=9606 GN=TDP1 PE=1 SV=2 | 0.87 |
| Q15007 | Pre-mRNA-splicing regulator WTAP OS=Homo sapiens OX=9606 GN=WTAP PE=1 SV=2 | 0.87 |
| Q9UK59 | Lariat debranching enzyme OS=Homo sapiens OX=9606 GN=DBR1 PE=1 SV=2 | 0.85 |
| P67809 | Y-box-binding protein 1 OS=Homo sapiens OX=9606 GN=YBX1 PE=1 SV=3 | 0.85 |
| P62753 | 40S ribosomal protein S6 OS=Homo sapiens OX=9606 GN=RPS6 PE=1 SV=1 | 0.85 |
| Q52LJ0 | Protein FAM98B OS=Homo sapiens OX=9606 GN=FAM98B PE=1 SV=2 | 0.81 |
| Q9BYJ9 | YTH domain-containing family protein 1 OS=Homo sapiens OX=9606 GN=YTHDF1 PE=1 SV=1 | 0.81 |
| P51513 | RNA-binding protein Nova-1 OS=Homo sapiens OX=9606 GN=NOVA1 PE=1 SV=2 | 0.8 |
| Q96FX7 | tRNA (adenine(58)-N(1))-methyltransferase catalytic subunit TRMT61A OS=Homo sapiens OX=9606 GN=TRMT61A PE=1 SV=1 | 0.77 |
| O00410 | Importin-5 OS=Homo sapiens OX=9606 GN=IPO5 PE=1 SV=4 | 0.76 |
| Q9BQ39 | ATP-dependent RNA helicase DDX50 OS=Homo sapiens OX=9606 GN=DDX50 PE=1 SV=1 | 0.76 |
| O43395 | U4/U6 small nuclear ribonucleoprotein Prp3 OS=Homo sapiens OX=9606 GN=PRPF3 PE=1 SV=2 | 0.75 |
| Q8TBF4 | Zinc finger CCHC-type and RNA-binding motif-containing protein 1 OS=Homo sapiens OX=9606 GN=ZCRB1 PE=1 SV=2 | 0.74 |
| O15116 | U6 snRNA-associated Sm-like protein LSm1 OS=Homo sapiens OX=9606 GN=LSM1 PE=1 SV=1 | 0.73 |
| O15347 | High mobility group protein B3 OS=Homo sapiens OX=9606 GN=HMGB3 PE=1 SV=4 | 0.64 |
| P16104 | Histone H2AX OS=Homo sapiens OX=9606 GN=H2AX PE=1 SV=2 | 0.64 |
| Q9BQ13 | BTB/POZ domain-containing protein KCTD14 OS=Homo sapiens OX=9606 GN=KCTD14 PE=1 SV=2 | 0.62 |
| Q8IUE6 | Histone H2A type 2-B OS=Homo sapiens OX=9606 GN=H2AC21 PE=1 SV=3 | 0.62 |
| P52435 | DNA-directed RNA polymerase II subunit RPB11-a OS=Homo sapiens OX=9606 GN=POLR2J PE=1 SV=1 | 0.61 |
| E5RJR5 | S-phase kinase-associated protein 1 OS=Homo sapiens OX=9606 GN=SKP1 PE=1 SV=1 | 0.59 |
| O00567 | Nucleolar protein 56 OS=Homo sapiens OX=9606 GN=NOP56 PE=1 SV=4 | 0.59 |
| Q15366 | Poly(rC)-binding protein 2 OS=Homo sapiens OX=9606 GN=PCBP2 PE=1 SV=1 | 0.57 |
| P43246 | DNA mismatch repair protein Msh2 OS=Homo sapiens OX=9606 GN=MSH2 PE=1 SV=1 | 0.56 |
| H0Y3P2 | Eukaryotic translation initiation factor 4 gamma 2 OS=Homo sapiens OX=9606 GN=EIF4G2 PE=1 SV=1 | 0.53 |
| Q9UKV8 | Protein argonaute-2 OS=Homo sapiens OX=9606 GN=AGO2 PE=1 SV=3 | 0.53 |
| P52701 | DNA mismatch repair protein Msh6 OS=Homo sapiens OX=9606 GN=MSH6 PE=1 SV=2 | 0.53 |
| Q15434 | RNA-binding motif, single-stranded-interacting protein 2 OS=Homo sapiens OX=9606 GN=RBMS2 PE=1 SV=1 | 0.51 |
| B4DHE8 | RNA-binding protein Musashi homolog 2 OS=Homo sapiens OX=9606 GN=MSI2 PE=1 SV=1 | 0.51 |
| P09651 | Heterogeneous nuclear ribonucleoprotein A1 OS=Homo sapiens OX=9606 GN=HNRNPA1 PE=1 SV=5 | 0.51 |
| S4R369 | 39S ribosomal protein L37, mitochondrial OS=Homo sapiens OX=9606 GN=MRPL37 PE=1 SV=1 | 0.5 |
| A0A087WY61 | Nuclear mitotic apparatus protein 1 OS=Homo sapiens OX=9606 GN=NUMA1 PE=1 SV=1 | 0.5 |
| O95104 | SR-related and CTD-associated factor 4 OS=Homo sapiens OX=9606 GN=SCAF4 PE=1 SV=3 | 0.5 |
| C9J2Y9 | DNA-directed RNA polymerase subunit beta OS=Homo sapiens OX=9606 GN=POLR2B PE=1 SV=2 | 0.49 |
| P06746 | DNA polymerase beta OS=Homo sapiens OX=9606 GN=POLB PE=1 SV=3 | 0.48 |
| P193882 | DNA-directed RNA polymerase II subunit E OS=Homo sapiens OX=9606 GN=POLR2E PE=1 SV= | 0.48 |
| Q86TB9 | Protein PAT1 homolog 1 OS=Homo sapiens OX=9606 GN=PATL1 PE=1 SV=2 | 0.48 |
| Q09028 | Histone-binding protein RBBP4 OS=Homo sapiens OX=9606 GN=RBBP4 PE=1 SV=3 | 0.48 |
| Q9UNZ5 | Leydig cell tumor 10 kDa protein homolog OS=Homo sapiens OX=9606 GN=C19orf53 PE=1 SV=1 | 0.48 |
| Q9BW19 | Kinesin-like protein KIFC1 OS=Homo sapiens OX=9606 GN=KIFC1 PE=1 SV=2 | 0.48 |
| Q9Y5A9 | YTH domain-containing family protein 2 OS=Homo sapiens OX=9606 GN=YTHDF2 PE=1 SV=2 | 0.47 |
| P19388 | DNA-directed RNA polymerases I, II, and III subunit RPABC1 OS=Homo sapiens OX=9606 GN=POLR2E PE=1 SV=4 | 0.47 |
| Q2NL82 | Pre-rRNA-processing protein TSR1 homolog OS=Homo sapiens OX=9606 GN=TSR1 PE=1 SV=1 | 0.46 |
| Q9Y3Y2 | Chromatin target of PRMT1 protein OS=Homo sapiens OX=9606 GN=CHTOP PE=1 SV=2 | 0.46 |
| Q1KMD3 | Heterogeneous nuclear ribonucleoprotein U-like protein 2 OS=Homo sapiens OX=9606 GN=HNRNPUL2 PE=1 SV=1 | 0.46 |
| Q9UK61 | Protein TASOR OS=Homo sapiens OX=9606 GN=TASOR PE=1 SV=3 | 0.46 |
| Q16666 | Gamma-interferon-inducible protein 16 OS=Homo sapiens OX=9606 GN=IFI16 PE=1 SV=3 | 0.46 |
| Q9H9Y6 | DNA-directed RNA polymerase I subunit RPA2 OS=Homo sapiens OX=9606 GN=POLR1B PE=1 SV=2 | 0.45 |
| F8W881 | Constitutive coactivator of PPAR-gamma-like protein 2 OS=Homo sapiens OX=9606 GN=FAM120C PE=1 SV=2 | 0.45 |
| P51149 | Ras-related protein Rab-7a OS=Homo sapiens OX=9606 GN=RAB7A PE=1 SV=1 | 0.44 |
| Q9H6F5 | Coiled-coil domain-containing protein 86 OS=Homo sapiens OX=9606 GN=CCDC86 PE=1 SV=1 | 0.44 |
| P40937 | Replication factor C subunit 5 OS=Homo sapiens OX=9606 GN=RFC5 PE=1 SV=1 | 0.44 |
| Q9NXV6 | CDKN2A-interacting protein OS=Homo sapiens OX=9606 GN=CDKN2AIP PE=1 SV=3 | 0.44 |
| Q6P6C2 | RNA demethylase ALKBH5 OS=Homo sapiens OX=9606 GN=ALKBH5 PE=1 SV=2 | 0.44 |
| P27816 | Microtubule-associated protein 4 OS=Homo sapiens OX=9606 GN=MAP4 PE=1 SV=3 | 0.43 |
| Q4TT34 | Nucleoside diphosphate kinase OS=Homo sapiens OX=9606 GN=NME4 PE=1 SV=1 | 0.43 |
| B9ZVN9 | DNA-directed RNA polymerase subunit OS=Homo sapiens OX=9606 GN=POLR1A PE=1 SV=1 | 0.43 |
| Q8NBJ5 | Procollagen galactosyltransferase 1 OS=Homo sapiens OX=9606 GN=COLGALT1 PE=1 SV=1 | 0.42 |
| Q9UNS2 | COP9 signalosome complex subunit 3 OS=Homo sapiens OX=9606 GN=COPS3 PE=1 SV=3 | 0.42 |
| Q5SW79 | Centrosomal protein of 170 kDa OS=Homo sapiens OX=9606 GN=CEP170 PE=1 SV=1 | 0.42 |
| P62841 | 40S ribosomal protein S15 OS=Homo sapiens OX=9606 GN=RPS15 PE=1 SV=2 | 0.42 |
| P26358 | DNA (cytosine-5)-methyltransferase 1 OS=Homo sapiens OX=9606 GN=DNMT1 PE=1 SV=2 | 0.41 |
| Q8IX12 | Cell division cycle and apoptosis regulator protein 1 OS=Homo sapiens OX=9606 GN=CCAR1 PE=1 SV=2 | 0.41 |
| P11940 | Polyadenylate-binding protein 1 OS=Homo sapiens OX=9606 GN=PABPC1 PE=1 SV=2 | 0.41 |
| Q14103 | Heterogeneous nuclear ribonucleoprotein D0 OS=Homo sapiens OX=9606 GN=HNRNPD PE=1 SV=1 | 0.41 |
| P09429 | High mobility group protein B1 OS=Homo sapiens OX=9606 GN=HMGB1 PE=1 SV=3 | 0.41 |
| Q13547 | Histone deacetylase 1 OS=Homo sapiens OX=9606 GN=HDAC1 PE=1 SV=1 | 0.41 |
| P11388 | DNA topoisomerase 2-alpha OS=Homo sapiens OX=9606 GN=TOP2A PE=1 SV=3 | 0.4 |
| Q8N684 | Cleavage and polyadenylation specificity factor subunit 7 OS=Homo sapiens OX=9606 GN=CPSF7 PE=1 SV=1 | 0.4 |
| P49750 | YLP motif-containing protein 1 OS=Homo sapiens OX=9606 GN=YLPM1 PE=1 SV=4 | 0.4 |
| Q5JVF3 | PCI domain-containing protein 2 OS=Homo sapiens OX=9606 GN=PCID2 PE=1 SV=2 | 0.39 |
| E7EW49 | CLIP-associating protein 2 OS=Homo sapiens OX=9606 GN=CLASP2 PE=1 SV=1 | 0.39 |
| P49458 | Signal recognition particle 9 kDa protein OS=Homo sapiens OX=9606 GN=SRP9 PE=1 SV=2 | 0.39 |
| F8W0Q9 | Periphilin-1 OS=Homo sapiens OX=9606 GN=PPHLN1 PE=1 SV=1 | 0.39 |
| Q5SSJ5 | Heterochromatin protein 1-binding protein 3 OS=Homo sapiens OX=9606 GN=HP1BP3 PE=1 SV=1 | 0.39 |
| Q9NRW3 | DNA dC->dU-editing enzyme APOBEC-3C OS=Homo sapiens OX=9606 GN=APOBEC3C PE=1 SV=2 | 0.39 |
| P19387 | DNA-directed RNA polymerase II subunit RPB3 OS=Homo sapiens OX=9606 GN=POLR2C PE=1 SV=2 | 0.38 |
| P62875 | DNA-directed RNA polymerases I, II, and III subunit RPABC5 OS=Homo sapiens OX=9606 GN=POLR2L PE=1 SV=2 | 0.38 |
| O14874 | [3-methyl-2-oxobutanoate dehydrogenase [lipoamide]] kinase, mitochondrial OS=Homo sapiens OX=9606 GN=BCKDK PE=1 SV=2 | 0.38 |
| O15160 | DNA-directed RNA polymerases I and III subunit RPAC1 OS=Homo sapiens OX=9606 GN=POLR1C PE=1 SV=1 | 0.38 |
| Q9BT43 | DNA-directed RNA polymerase subunit RPC7 OS=Homo sapiens OX=9606 GN=POLR3GL PE=1 SV=2 | 0.38 |
| O95758 | Polypyrimidine tract-binding protein 3 OS=Homo sapiens OX=9606 GN=PTBP3 PE=1 SV=2 | 0.38 |
| P24928 | DNA-directed RNA polymerase II subunit RPB1 OS=Homo sapiens OX=9606 GN=POLRA2 PE=1 SV=2 | 0.38 |
| Q96QC0 | Serine/threonine-protein phosphatase 1 regulatory subunit 10 OS=Homo sapiens OX=9606 GN=PPP1R10 PE=1 SV=1 | 0.38 |
| Q9BQ04 | RNA-binding protein 4B OS=Homo sapiens OX=9606 GN=RBM4B PE=1 SV=1 | 0.38 |
| A0A087X2B6 | Cell cycle and apoptosis regulator protein 2 OS=Homo sapiens OX=9606 GN=CCAR2 PE=1 SV=1 | 0.37 |
| Q96E39 | RNA binding motif protein, X-linked-like-1 OS=Homo sapiens OX=9606 GN=RBMXL1 PE=1 SV=1 | 0.37 |
| O14979 | Heterogeneous nuclear ribonucleoprotein D-like OS=Homo sapiens OX=9606 GN=HNRNPDL PE=1 SV=3 | 0.36 |
| F8W8I6 | Nucleolysin TIA-1 isoform p40 OS=Homo sapiens OX=9606 GN=TIA1 PE=1 SV=1 | 0.36 |
| P16402 | Histone H1.3 OS=Homo sapiens OX=9606 GN=H1-3 PE=1 SV=2 | 0.36 |
| Q05048 | Cleavage stimulation factor subunit 1 OS=Homo sapiens OX=9606 GN=CSTF1 PE=1 SV=1 | 0.36 |
| A0A1C7CYX1 | Mitotic deacetylase-associated SANT domain protein OS=Homo sapiens OX=9606 GN=MIDEAS PE=1 SV=1 | 0.36 |
| Q92576 | PHD finger protein 3 OS=Homo sapiens OX=9606 GN=PHF3 PE=1 SV=3 | 0.36 |
| A0A3B3IU51 | Tight junction protein ZO-2 OS=Homo sapiens OX=9606 GN=TJP2 PE=1 SV=1 | 0.36 |
| Q9Y520 | Protein PRRC2C OS=Homo sapiens OX=9606 GN=PRRC2C PE=1 SV=4 | 0.35 |
| O60930 | Ribonuclease H1 OS=Homo sapiens OX=9606 GN=RNASEH1 PE=1 SV=2 | 0.35 |
| Q96GD4 | Aurora kinase B OS=Homo sapiens OX=9606 GN=AURKB PE=1 SV=3 | 0.35 |
| Q9P0W2 | SWI/SNF-related matrix-associated actin-dependent regulator of chromatin subfamily E member 1-related OS=Homo sapiens OX=9606 GN=HMG20B PE=1 SV=1 | 0.35 |
| G3V167 | Poly [ADP-ribose] polymerase OS=Homo sapiens OX=9606 GN=PARP2 PE=1 SV=1 | 0.34 |
| E7EVE9 | General transcription and DNA repair factor IIH helicase subunit XPD OS=Homo sapiens OX=9606 GN=ERCC2 PE=1 SV=1 | 0.33 |
| P17676 | CCAAT/enhancer-binding protein beta OS=Homo sapiens OX=9606 GN=CEBPB PE=1 SV=3 | 0.32 |
| Q9P2N5 | RNA-binding protein 27 OS=Homo sapiens OX=9606 GN=RBM27 PE=1 SV=2 | 0.31 |
| P33240 | Cleavage stimulation factor subunit 2 OS=Homo sapiens OX=9606 GN=CSTF2 PE=1 SV=1 | 0.3 |
| P46013 | Proliferation marker protein Ki-67 OS=Homo sapiens OX=9606 GN=MKI67 PE=1 SV=2 | 0.29 |
| Q9H2U1 | ATP-dependent DNA/RNA helicase DHX36 OS=Homo sapiens OX=9606 GN=DHX36 PE=1 SV=2 | 0.29 |
| P27695 | DNA-(apurinic or apyrimidinic site) endonuclease OS=Homo sapiens OX=9606 GN=APEX1 PE=1 SV=2 | 0.29 |
| Q00577 | Transcriptional activator protein Pur-alpha OS=Homo sapiens OX=9606 GN=PURA PE=1 SV=2 | 0.28 |
| P48634 | Protein PRRC2A OS=Homo sapiens OX=9606 GN=PRRC2A PE=1 SV=3 | 0.26 |
| Q9ULW0 | Targeting protein for Xklp2 OS=Homo sapiens OX=9606 GN=TPX2 PE=1 SV=2 | 0.26 |
| P14866 | Heterogeneous nuclear ribonucleoprotein L OS=Homo sapiens OX=9606 GN=HNRNPL PE=1 SV=2 | 0.25 |
| Q96I25 | Splicing factor 45 OS=Homo sapiens OX=9606 GN=RBM17 PE=1 SV=1 | 0.24 |
| P50213 | Isocitrate dehydrogenase [NAD] subunit alpha, mitochondrial OS=Homo sapiens OX=9606 GN=IDH3A PE=1 SV=1 | 0.24 |
| Q14011 | Cold-inducible RNA-binding protein OS=Homo sapiens OX=9606 GN=CIRBP PE=1 SV=1 | 0.23 |
| Q9BQP7 | Mitochondrial genome maintenance exonuclease 1 OS=Homo sapiens OX=9606 GN=MGME1 PE=1 SV=1 | 0.23 |
| O75525 | KH domain-containing, RNA-binding, signal transduction-associated protein 3 OS=Homo sapiens OX=9606 GN=KHDRBS3 PE=1 SV=1 | 0.21 |
| H0Y993 | DEK oncogene (DNA binding), isoform CRA_b OS=Homo sapiens OX=9606 GN=DEK PE=1 SV=2 | 0.21 |
| Q8WWM7 | Ataxin-2-like protein OS=Homo sapiens OX=9606 GN=ATXN2L PE=1 SV=2 | 0.2 |
| Q15637 | Splicing factor 1 OS=Homo sapiens OX=9606 GN=SF1 PE=1 SV=4 | 0.18 |
| Q8WXF1 | Paraspeckle component 1 OS=Homo sapiens OX=9606 GN=PSPC1 PE=1 SV=1 | 0.18 |
| Q2NKJ3 | CST complex subunit CTC1 OS=Homo sapiens OX=9606 GN=CTC1 PE=1 SV=2 | 0.17 |
| P62140 | Serine/threonine-protein phosphatase PP1-beta catalytic subunit OS=Homo sapiens OX=9606 GN=PPP1CB PE=1 SV=3 | 0.17 |
| P49916 | DNA ligase 3 OS=Homo sapiens OX=9606 GN=LIG3 PE=1 SV=2 | 0.13 |
| A0A087WZ13 | Ribonucleoprotein PTB-binding 1 OS=Homo sapiens OX=9606 GN=RAVER1 PE=1 SV=1 | 0.13 |
| Q9H0D6 | 5'-3' exoribonuclease 2 OS=Homo sapiens OX=9606 GN=XRN2 PE=1 SV=1 | 0.12 |
| F5H101 | Nucleolar protein 8 OS=Homo sapiens OX=9606 GN=NOL8 PE=1 SV=1 | 0.12 |
| Q15650 | Activating signal cointegrator 1 OS=Homo sapiens OX=9606 GN=TRIP4 PE=1 SV=4 | 0.12 |
| Q92466 | DNA damage-binding protein 2 OS=Homo sapiens OX=9606 GN=DDB2 PE=1 SV=1 | 0.12 |
| Q96HQ2 | CDKN2AIP N-terminal-like protein OS=Homo sapiens OX=9606 GN=CDKN2AIPNL PE=1 SV=1 | 0.11 |
| Q99590 | Protein SCAF11 OS=Homo sapiens OX=9606 GN=SCAF11 PE=1 SV=2 | 0.11 |
| Q9UKK6 | NTF2-related export protein 1 OS=Homo sapiens OX=9606 GN=NXT1 PE=1 SV=1 | 0.11 |
| Q03701 | CCAAT/enhancer-binding protein zeta OS=Homo sapiens OX=9606 GN=CEBPZ PE=1 SV=3 | 0.11 |
| Q96QR8 | Transcriptional activator protein Pur-beta OS=Homo sapiens OX=9606 GN=PURB PE=1 SV=3 | 0.1 |
| Q8NCA5 | Protein FAM98A OS=Homo sapiens OX=9606 GN=FAM98A PE=1 SV=2 | 0.1 |
| Q9NX24 | H/ACA ribonucleoprotein complex subunit 2 OS=Homo sapiens OX=9606 GN=NHP2 PE=1 SV=1 | 0.1 |
| O94762 | ATP-dependent DNA helicase Q5 OS=Homo sapiens OX=9606 GN=RECQL5 PE=1 SV=2 | 0.09 |
| Q9NY12 | H/ACA ribonucleoprotein complex subunit 1 OS=Homo sapiens OX=9606 GN=GAR1 PE=1 SV=1 | 0.09 |
| A0A5F9ZHP1 | Activating signal cointegrator 1 complex subunit 1 OS=Homo sapiens OX=9606 GN=ASCC1 PE=1 SV=1 | 0.09 |
| Q01831 | DNA repair protein complementing XP-C cells OS=Homo sapiens OX=9606 GN=XPC PE=1 SV=4 | 0.09 |
| Q96KR1 | Zinc finger RNA-binding protein OS=Homo sapiens OX=9606 GN=ZFR PE=1 SV=2 | 0.08 |
| Q9H147 | Deoxynucleotidyltransferase terminal-interacting protein 1 OS=Homo sapiens OX=9606 GN=DNTTIP1 PE=1 SV=2 | 0.07 |
| O94842 | TOX high mobility group box family member 4 OS=Homo sapiens OX=9606 GN=TOX4 PE=1 SV=1 | 0.07 |
| Q8WVV9 | Heterogeneous nuclear ribonucleoprotein L-like OS=Homo sapiens OX=9606 GN=HNRNPLL PE=1 SV=1 | 0.07 |
| Q96NB3 | Zinc finger protein 830 OS=Homo sapiens OX=9606 GN=ZNF830 PE=1 SV=2 | 0.06 |
| P0DMU9 | Cancer/testis antigen family 45 member A10 OS=Homo sapiens OX=9606 GN=CT45A10 PE=1 SV=1 | 0.06 |
| O60832 | H/ACA ribonucleoprotein complex subunit DKC1 OS=Homo sapiens OX=9606 GN=DKC1 PE=1 SV=3 | 0.06 |
| P54727 | UV excision repair protein RAD23 homolog B OS=Homo sapiens OX=9606 GN=RAD23B PE=1 SV=1 | 0.06 |
| Q13472 | DNA topoisomerase 3-alpha OS=Homo sapiens OX=9606 GN=TOP3A PE=1 SV=1 | 0.05 |
| P78332 | RNA-binding protein 6 OS=Homo sapiens OX=9606 GN=RBM6 PE=1 SV=5 | 0.05 |
| P29372 | DNA-3-methyladenine glycosylase OS=Homo sapiens OX=9606 GN=MPG PE=1 SV=3 | 0.05 |
| Q08426 | Peroxisomal bifunctional enzyme OS=Homo sapiens OX=9606 GN=EHHADH PE=1 SV=3 | 0.05 |
| Q9H9A7 | RecQ-mediated genome instability protein 1 OS=Homo sapiens OX=9606 GN=RMI1 PE=1 SV=3 | 0.05 |
| P54098 | DNA polymerase subunit gamma-1 OS=Homo sapiens OX=9606 GN=POLG PE=1 SV=1 | 0.04 |
| P20585 | DNA mismatch repair protein Msh3 OS=Homo sapiens OX=9606 GN=MSH3 PE=1 SV=4 | 0.04 |
| P78549 | Endonuclease III-like protein 1 OS=Homo sapiens OX=9606 GN=NTHL1 PE=1 SV=2 | 0.04 |
| Q8N5C6 | S1 RNA-binding domain-containing protein 1 OS=Homo sapiens OX=9606 GN=SRBD1 PE=1 SV=2 | 0.04 |
| P41208 | Centrin-2 OS=Homo sapiens OX=9606 GN=CETN2 PE=1 SV=1 | 0.04 |
| Q14527 | Helicase-like transcription factor OS=Homo sapiens OX=9606 GN=HLTF PE=1 SV=2 | 0.03 |
| Q9NPE3 | H/ACA ribonucleoprotein complex subunit 3 OS=Homo sapiens OX=9606 GN=NOP10 PE=1 SV=1 | 0.01 |
| C9JIJ9 | RNA-binding motif, single-stranded-interacting protein 3 OS=Homo sapiens OX=9606 GN=RBMS3 PE=1 SV=1 | 0.01 |
| Q9NRY2 | SOSS complex subunit C OS=Homo sapiens OX=9606 GN=INIP PE=1 SV=1 | 0.01 |
| P54725 | UV excision repair protein RAD23 homolog A OS=Homo sapiens OX=9606 GN=RAD23A PE=1 SV=1 | 0.01 |
| Q86V81 | THO complex subunit 4 OS=Homo sapiens OX=9606 GN=ALYREF PE=1 SV=3 | 0.01 |
