# Supplementary material for: rs822336 binding to C/EBPβ and NFIC modulates induction of PD-L1 expression and predicts anti-PD-1/PD-L1 therapy in advanced NSCLC
Source: Mol Cancer. 2024 Mar 25;23:63. doi: 10.1186/s12943-024-01976-2 (PMC10962156; doi:10.1186/s12943-024-01976-2)

**Figure S8** Silencing of C/EBPβ and NFIC in EGFR^mut^ H1975^G/G^ and EGFR^wt^ H1299^C/C^ cell lines. Cells were seeded into 6-well plates at a density of 2×10^6^ cells per well. Following a 48h of transfection at 37°C in a 5% CO_2_ atmosphere with C/EBPβ - and NFIC-specific siRNAs cells were harvested and lysed. A siRNA-control was used as a control. Cell lysates were analyzed by western blot with C/EBPβ and NFIC-specific Abs. GAPDH was used as a loading control. Representative results are shown.


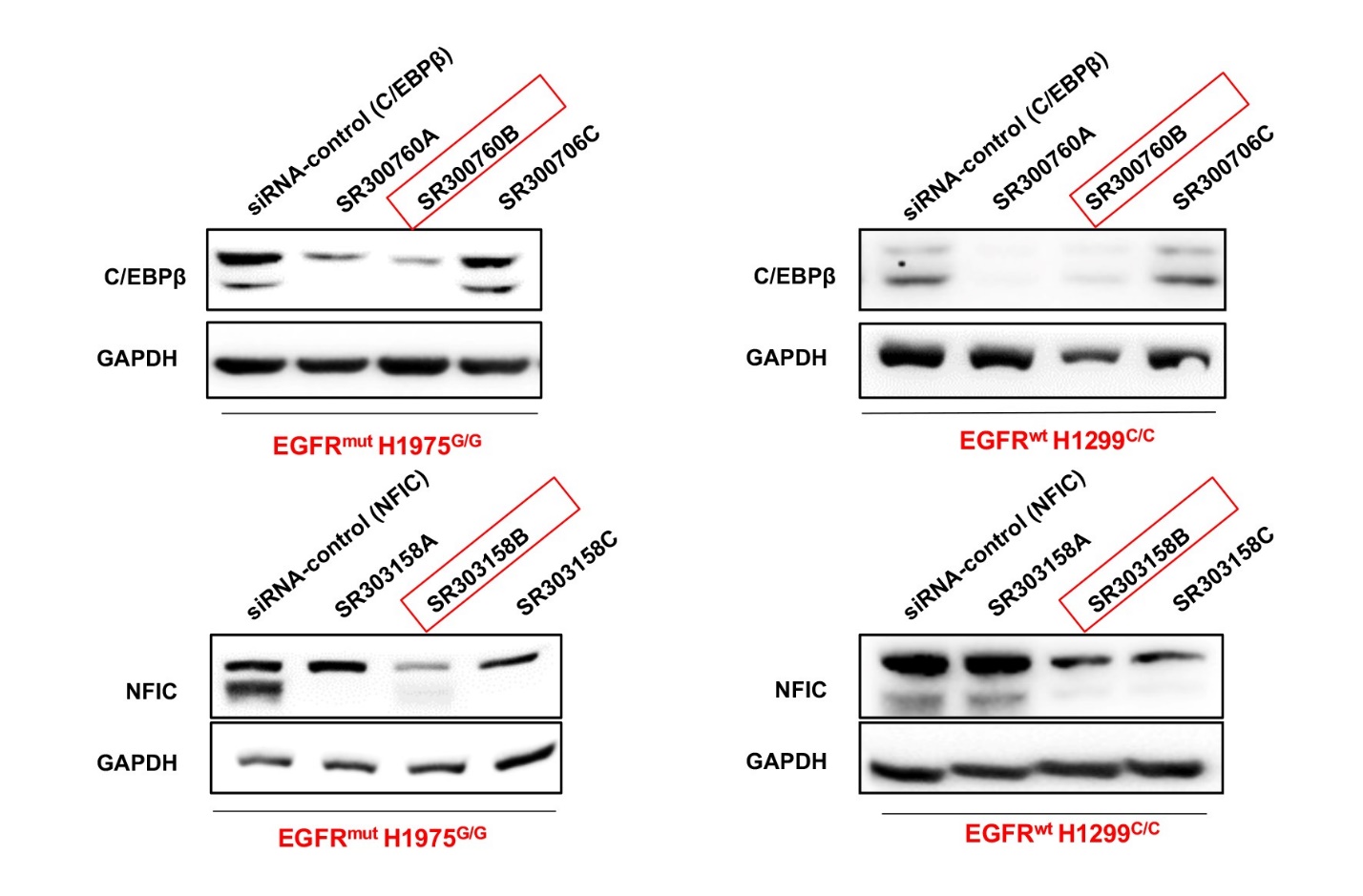

Supplement: Supplementary file 10 — Additional file 10: figure S8 Silencing of C/EBPβ and NFIC in EGFRmut H1975G/G and EGFRwt H1299C/C cell lines. Cells were seeded into 6-well plates at a density of 2 × 10^6^ cells per well. Following a 48 h of transfection at 37 °C in a 5% CO2 atmosphere with C/EBPβ - and NFIC-specific siRNAs cells were harvested and lysed. A siRNA-control was used as a control. Cell lysates were analyzed by western blot with C/EBPβ and NFIC-specific Abs. GAPDH was used as a loading control. Representative results are shown. [file 12943_2024_1976_MOESM10_ESM.docx]
