# Supplementary material for: rs822336 binding to C/EBPβ and NFIC modulates induction of PD-L1 expression and predicts anti-PD-1/PD-L1 therapy in advanced NSCLC
Source: Mol Cancer. 2024 Mar 25;23:63. doi: 10.1186/s12943-024-01976-2 (PMC10962156; doi:10.1186/s12943-024-01976-2)

**Figure S9** EGFR^mut^ H1975^G/G^ and EGFR^wt^ H1299^C/C^ cells transduced with C/EBPβ- and NFIC-specific siRNAs or siRNA-controls were seeded into 24-well plates at a density of 2×10^5^ cells per well and incubated with IFN-ɣ (100ng/ml). Untreated cells were used as a control. Following a 24h incubation at 37°C in a 5% CO_2_ atmosphere, expression levels of NFIC, C/EBPβ and PD-L1 mRNA were evaluated by Real-Time (RT)-PCR. The levels of NFIC, C/EBPβ and PD-L1, normalized to GAPDH and relative to untreated siRNA-control of each analyzed gene, are plotted and expressed as mean ± SD of the results obtained in three independent experiments (***P ≤ 0.001).


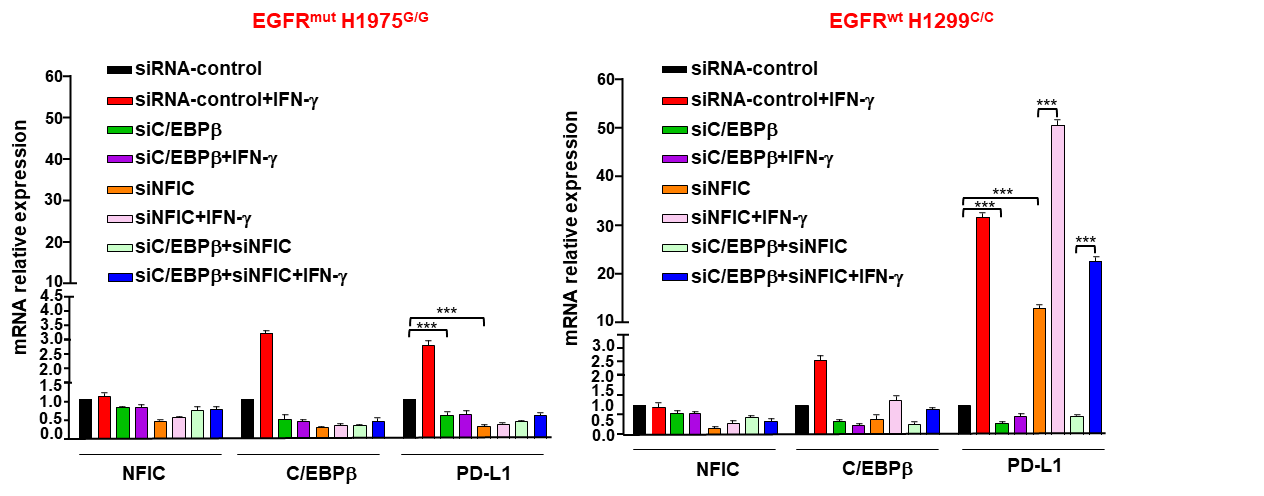

Supplement: Supplementary file 11 — Additional file 11: figure S9 EGFRmut H1975G/G and EGFRwt H1299C/C cells transduced with C/EBPβ- and NFIC-specific siRNAs or siRNA-controls were seeded into 24-well plates at a density of 2 × 10^5^ cells per well and incubated with IFN-ɣ (100ng/ml). Untreated cells were used as a control. Following a 24 h incubation at 37 °C in a 5% CO2 atmosphere, expression levels of NFIC, C/EBPβ and PD-L1 mRNA were evaluated by Real-Time (RT)-PCR. The levels of NFIC, C/EBPβ and PD-L1, normalized to GAPDH and relative to untreated siRNA-control of each analysed gene, are plotted and expressed as mean ± SD of the results obtained in three independent experiments (***P ≤ 0.001). [file 12943_2024_1976_MOESM11_ESM.docx]
