# Supplementary material for: rs822336 binding to C/EBPβ and NFIC modulates induction of PD-L1 expression and predicts anti-PD-1/PD-L1 therapy in advanced NSCLC
Source: Mol Cancer. 2024 Mar 25;23:63. doi: 10.1186/s12943-024-01976-2 (PMC10962156; doi:10.1186/s12943-024-01976-2)

**Figure S10** Activation of IFN-ɣ pathway in NSCLC cell lines transfected with siRNAs. EGFR^mut^ H1975^G/G^ (left panel) and EGFR^wt^ H1299^C/C^ (right panel) cells were seeded into 6-well plates at a density of 2×10^6^ cells per well and incubated with IFN-ɣ (100ng/ml). Untreated cells were used as a control. Following a 48h of incubation at 37°C in a 5% CO_2_ atmosphere, cells transfected with the indicated siRNAs were harvested and lysed. Cell lysates were analyzed by western blot with p-STAT1 and STAT1-specific Abs. Representative results are shown. The levels of STAT1, normalized to GAPDH and relative to untreated siRNA-control as well as levels of p-STAT1 normalized to STAT1, are plotted below and expressed as mean ± SD of the results obtained in three independent experiments (***P ≤ 0.001).


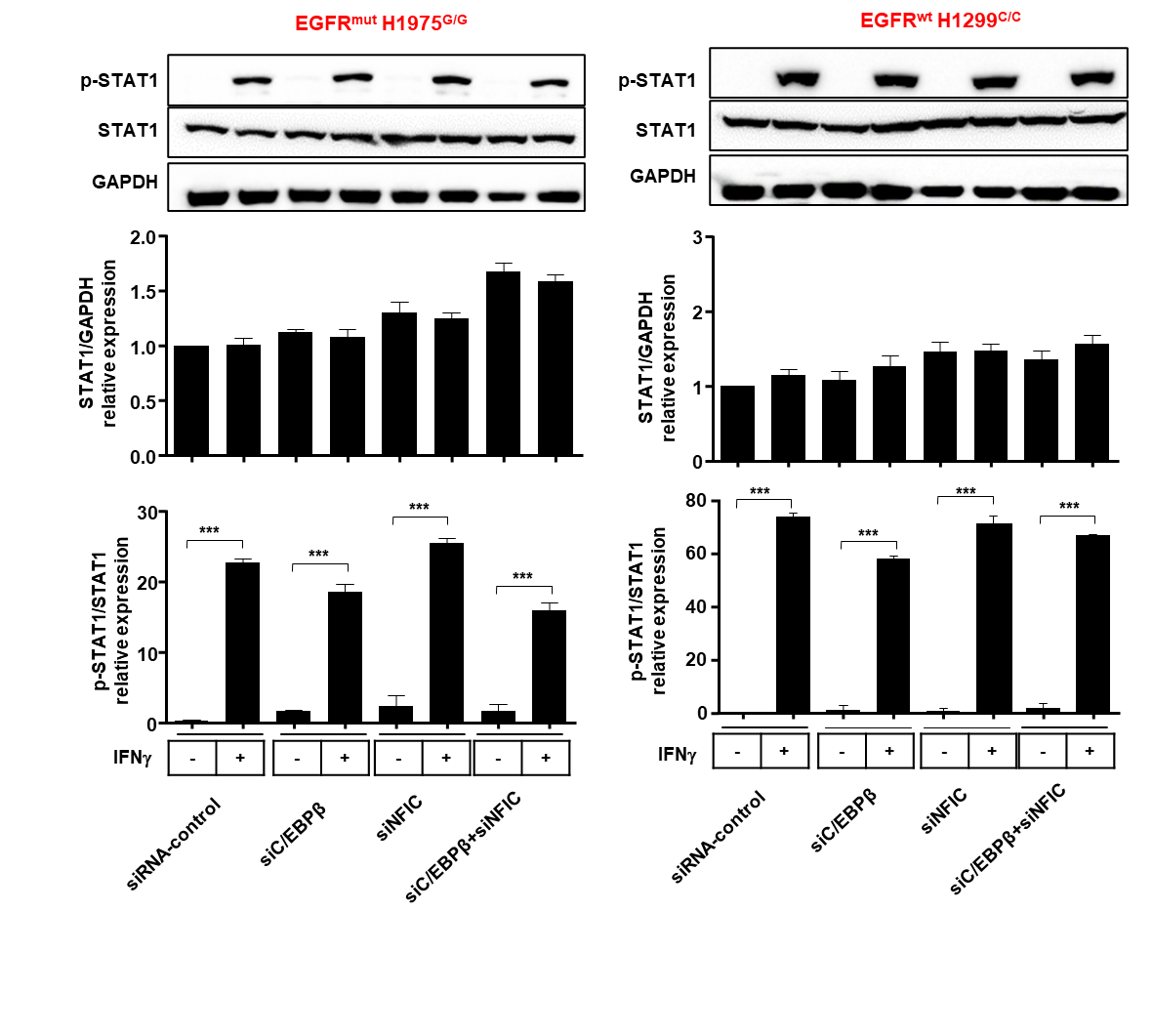

Supplement: Supplementary file 12 — Additional file 12: figure S10 Activation of IFN-ɣ pathway in NSCLC cell lines transfected with siRNAs. EGFRmut H1975G/G (left panel) and EGFRwt H1299C/C (right panel) cells were seeded into 6-well plates at a density of 2 × 10^6^ cells per well and incubated with IFN-ɣ (100ng/ml). Untreated cells were used as a control. Following a 48 h of incubation at 37 °C in a 5% CO2 atmosphere, cells transfected with the indicated siRNAs were harvested and lysed. Cell lysates were analyzed by western blot with p-STAT1 and STAT1-specific Abs. Representative results are shown. The levels of STAT1, normalized to GAPDH and relative to untreated siRNA-control as well as levels of p-STAT1 normalized to STAT1, are plotted below and expressed as mean ± SD of the results obtained in three independent experiments (***P ≤ 0.001). [file 12943_2024_1976_MOESM12_ESM.docx]
